# Atypical cholangiocytes derived from hepatocyte-cholangiocyte transdifferentiation mediated by COX-2: a kind of misguided liver regeneration

**DOI:** 10.1186/s41232-023-00284-4

**Published:** 2023-07-14

**Authors:** Tian Lan, Yang Tai, Chong Zhao, Yang Xiao, Zhu Yang, Linhao Zhang, Can Gan, Wenting Dai, Huan Tong, Chengwei Tang, Zhiyin Huang, Jinhang Gao

**Affiliations:** 1 Laboratory of Gastroenterology and Hepatology, State Key Laboratory of Biotherapy, West China Hospital, Sichuan University, Chengdu, 610041 China; 2 Department of Gastroenterology, West China Hospital, Sichuan University, Chengdu, 610041 China

**Keywords:** Hepatocyte-cholangiocyte transdifferentiation, Cyclooxygenase-2, β-catenin, TGF-β, Chronic liver injury, Liver regeneration

## Abstract

**Background:**

Hepatocyte-cholangiocyte transdifferentiation (HCT) is a potential origin of proliferating cholangiocytes in liver regeneration after chronic injury. This study aimed to determine HCT after chronic liver injury, verify the impacts of HCT on liver repair, and avoid harmful regeneration by understanding the mechanism.

**Methods:**

A thioacetamide (TAA)-induced liver injury model was established in wild-type (WT-TAA group) and COX-2 panknockout (KO-TAA group) mice. HCT was identified by costaining of hepatocyte and cholangiocyte markers in vivo and in isolated mouse hepatocytes in vitro. The biliary tract was injected with ink and visualized by whole liver optical clearing. Serum and liver bile acid (BA) concentrations were measured. Either a COX-2 selective inhibitor or a β-catenin pathway inhibitor was administered in vitro.

**Results:**

Intrahepatic ductular reaction was associated with COX-2 upregulation in chronic liver injury. Immunofluorescence and RNA sequencing indicated that atypical cholangiocytes were characterized by an intermediate genetic phenotype between hepatocytes and cholangiocytes and might be derived from hepatocytes. The structure of the biliary system was impaired, and BA metabolism was dysregulated by HCT, which was mediated by the TGF-β/β-catenin signaling pathway. Genetic deletion or pharmaceutical inhibition of COX-2 significantly reduced HCT in vivo. The COX-2 selective inhibitor etoricoxib suppressed HCT through the TGF-β-TGFBR1-β-catenin pathway in vitro.

**Conclusions:**

Atypical cholangiocytes can be derived from HCT, which forms a secondary strike by maldevelopment of the bile drainage system and BA homeostasis disequilibrium during chronic liver injury. Inhibition of COX-2 could ameliorate HCT through the COX-2-TGF-β-TGFBR1-β-catenin pathway and improve liver function.

**Supplementary Information:**

The online version contains supplementary material available at 10.1186/s41232-023-00284-4.

## Introduction

The liver parenchyma, comprised of hepatocytes and cholangiocytes, accounts for 70–80% of the total liver mass and maintains hepatic homeostasis [1]. Under normal conditions, hepatocytes and cholangiocytes maintain a low turnover rate to compensate for occasional cell loss, such as apoptosis and senescence [1, 2]. The unique regenerative capacity of the liver in acute injury is always beneficial and has been extensively studied [3]. However, when insulted by prolonged chronic injury, the exhausted regeneration potential of liver parenchymal cells is usually insufficient to complete tissue repair only by their own mitosis and proliferation. The transdifferentiation between hepatocytes and cholangiocytes has been observed [4]. Proliferation of cholangiocytes, also known as ductular reaction (DR), is commonly presented in liver regeneration from chronic injury [5]. Unlike hepatocyte mitosis, the cholangiocytes in DR may originate from putative hepatic progenitor cells (HPCs), which are difficult to determine in human tissue studies [6]. In the study of cholestatic liver diseases, hepatocyte-cholangiocyte transdifferentiation (HCT), a process in which hepatocytes transdifferentiate into cholangiocytes, was described [7]. However, the origin of cholangiocytes in DR due to chronic hepatocytic injuries, such as hepatitis B virus (HBV) infection, nonalcoholic steatohepatitis (NASH), and alcoholic liver disease (ALD), remains unclear. In addition, there are few studies regarding the actual effects of HCT on liver structural regeneration and functional repair in chronic liver injury. It is uncertain whether HCT is beneficial or detrimental to the liver. Theoretically, loss of hepatocytes due to HCT might lead to dysregulation of liver metabolism because hepatocytes exert a critical role in the synthesis and metabolism of proteins, glucose, lipids, and bile acids (BAs).

Cyclooxygenase-2 (COX-2) is a highly conserved and critical enzyme in prostanoid biosynthesis [8]. As an immediate-early response and highly inducible gene, upregulation of COX-2 has been reported in various inflammation or tumors [9]. Normally, less COX-2 expression is detected in the liver, but it is significantly increased in chronic liver injury, fibrosis, and cirrhosis, indicating the important role of COX-2 [9–11]. Genetic or pharmaceutical inhibition of COX-2 may ameliorate chronic liver injury and cirrhotic progression by reducing intrahepatic angiogenesis, inducing hepatic stellate cell apoptosis, and protecting sinusoidal function [12–15]. To date, there is no association study on intrahepatic COX-2 expression and HCT.

Mechanistically, the Hippo-YAP pathway, Notch-HES1 pathway, and Wnt-β-catenin pathway are involved in mediating HCT during biliary injury [16–19]. If HCT in liver injury could be regulated by COX-2, the underlying mechanism may learn from the transforming growth factor beta (TGF-β)/SMAD signaling pathway because COX-2 inhibitor intervened in epithelial-to-mesenchymal transition via this pathway in our previous study [15].

Therefore, this study aimed to determine the association of DR and COX-2 expression during chronic liver injury, whether cholangiocytes in DR originated from HCT, the impacts of HCT on liver structural regeneration and functional repair, and the involvement of the TGF-β-TGFBR1 or β-catenin signaling pathway in HCT mediated by COX-2. These elucidations may be helpful in preventing liver depletion from harmful liver regeneration.

## Materials and methods

### Human samples

For human liver tissue analyses, human liver explants were obtained from 11 patients with HBV cirrhosis at the time of liver transplant surgery. Control samples were collected from 7 patients receiving hepatic hemangioma resection with no chronic liver disease. Clinical information of these patients is provided in Supplementary Table S1. This study was approved by West China Hospital, Sichuan University, and was registered on the Chinese Clinical Trial Registry (http://www.chictr.org.cn/index.aspx, registering no. ChiCTR2200063108). Written informed consent forms were received from all involved patients. All procedures of this work were carried out following the guidelines and regulations of the institutional and national ethics committee.

### Animal experiments

Animal experiments were approved by the Animal Use and Nursing Committee of Sichuan University. Wild-type C57BL/6 mice and *Ella*^Cre^, *Alb*^Cre^, and *Cox-2*^flox/flox^ mice were purchased from GemPharmatech (Nanjing, China). Mice were kept in an environment of constant temperature and humidity with a 12-h:12-h light–dark cycle and free access to a chow diet and drinking water. *Ella*^Cre^ mice and *Alb*^Cre^ mice were crossed with *Cox-2*^flox/flox^ mice to generate *Cox-2* panknockout mice (*Ella*^*Cre*^*Cox-2*^flox/flox^) and hepatocyte-specific *Cox-2* knockout mice (*Alb*^Cre^*Cox-2*^flox/flox^), respectively. Age-matched male mice (6–8 weeks old) were selected for experiments. Wild-type mice (WT-TAA group), *Cox-2* panknockout mice (KO-TAA group), and hepatocyte-specific *Cox-2* knockout mice (CKO-TAA group) were injected intraperitoneally with 200 mg/kg TAA (Sigma‒Aldrich, St. Louis, MO, USA) every 3 days for 8 weeks to induce chronic liver injury. One group of wild-type mice was injected with TAA for 12 weeks and fed imrecoxib (20 mg/kg/day) by gastric gavage during the last 8 weeks (WT-TAA + IMRE group). Wild-type mice were injected with an equal volume of normal saline as the control (WT-NS group). The mice were anesthetized and sacrificed 24 h after the last injection. Liver and blood samples (collected from the orbital sinus at the time of sacrifice) were harvested for further experiments. A total of 0.1% DDC diet for 4 weeks and BDL for 4 weeks were also applied to induce chronic liver injury in mice.

### Statistical analysis

All quantitative data are expressed as the mean ± standard deviation. Independent-sample *t*-test with Welch’s test was applied to compare the two groups. One-way analysis of variance (ANOVA) was applied for multiple group comparisons. All statistical analyses were conducted using SPSS 19.0 software, and statistical significance was considered when *p* < 0.05. The remaining materials and methods are described in the supplementary file.

## Results

### Intrahepatic ductular reaction was associated with COX-2 upregulation in chronic liver injury

**Fig. 1:**
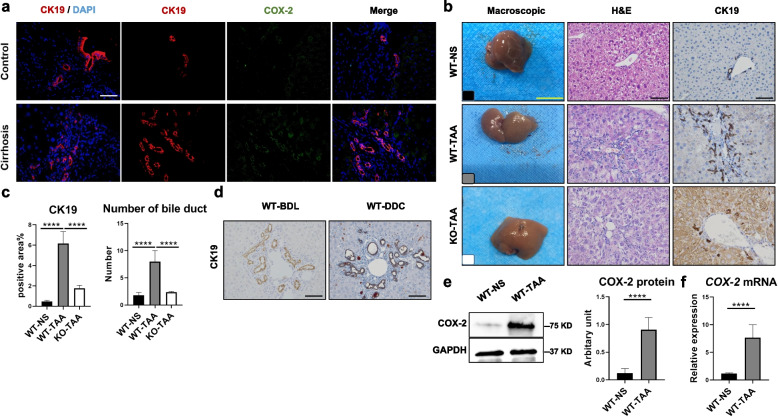
Intrahepatic atypical cholangiocyte proliferation was associated with COX-2 upregulation in cirrhotic patients and chronic hepatic injuries in mice. **a** Ductular reaction marked with CK19 and COX-2 in the liver tissue sections of patients with HBV cirrhosis (IF). **b** Ductular reaction marked with CK19 (IHC) in TAA-treated mice was reduced with COX-2 panknockout. **c** The CK19-positive area and number of bile ducts in the WT-NS, WT-TAA, and KO-TAA groups were measured. ****p < 0.0001 (one-way ANOVA, *n* = 6/group). **d** Ductular reaction marked with CK19 in the BDL and DDC models (IHC). **e** and **f** COX-2 expression in the livers of the WT-NS and WT-TAA groups was measured by WB and qPCR. Images were cropped and scaled for illustration purposes from original Western blot images provided in supplementary files. Black and white Scale bars 50μm, yellow scale bar 1cm. ****p < 0.0001 (*t*-test, *n* = 6/group)

The increased cholangiocyte marker cytokeratin 19 (CK19) localized at intrahepatic biliary ducts was detected in the liver tissue sections of 11 patients with HBV cirrhosis (Fig. 1a). Similarly, proliferated cholangiocyte-like cells marked with CK19 could also be seen in the tissue sections of mice treated with thioacetamide (TAA, Fig. 1b). The CK19-positive area and the number of bile duct lumens in the portal areas were significantly increased in the wild-type mice treated with TAA (WT-TAA group) compared to the control mice treated with normal saline (WT-NS group, Fig. 1c). In addition, the proliferating cholangiocytes in the WT-TAA group presented atypical cord-like arrangements with oval shapes and indistinguishable lumens (Fig. 1b), which were different from those of cholangiocytes in the 3,5-diethoxycarbonyl-1,4-dihydrocollidine (DDC) diet and bile duct ligation mouse models (BDL, Fig. 1d). Moreover, upregulation of COX-2 in cholangiocyte-like cells was observed not only in human cirrhotic livers (Fig. 1a) but also in mouse livers treated with TAA (Fig. 1e). The protein and mRNA levels of COX-2 were significantly higher in the WT-TAA group than in the WT-NS group (Fig. 1e, f). The cholangiocyte reaction due to chronic liver injury could be attenuated in mice with COX-2 panknockout (Fig. 1b, c).

### The atypical cholangiocytes were characterized by intermediate phenotype between hepatocytes and cholangiocytes and might be derived from hepatocytes

**Fig. 2:**
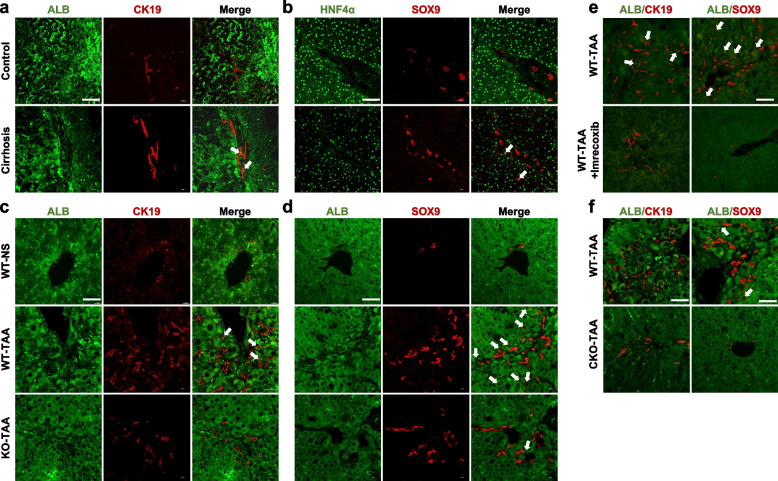
Visualization of atypical cholangiocyte proliferation derived from HCT in chronic liver injury. **a** and **b** Liver samples were collected from healthy controls and patients with liver cirrhosis. HCT was measured by IF of hepatocyte markers (ALB, HNF4α) and cholangiocyte markers (CK19, SOX9). **c** and **d** HCT was measured in livers of the WT-NS, WT-TAA, and KO-TAA groups by IF of hepatocyte marker (ALB) and cholangiocyte markers (CK19, SOX9). **e** and **f** HCT was measured by IF of hepatocyte marker (ALB) and cholangiocyte markers (CK19, SOX9) in livers of the WT-TAA and WT-TAA + imrecocxib groups (**e**) and in the WT-TAA and CKO-TAA groups (**f**). White arrows indicate coexpression or colocalization of hepatocyte and cholangiocyte markers. Scale bars 50μm

The atypical cholangiocytes in the liver tissue sections of chronic liver injury were costained with the hepatocyte markers albumin (ALB) and hepatocyte nuclear factor 4α (HNF4α) in combination with the cholangiocyte markers CK19 and SRY (sex determining region Y)-box 9 (SOX9). There was no colocalization of ALB and cholangiocyte markers in the livers of the control group of either humans or mice. However, immunofluorescence (IF) showed that ALB and cholangiocyte markers were colocalized in the intrahepatic atypical bile ducts of either human cirrhosis or mice treated with TAA (Fig. 2a–d), suggesting that the intermediate phenotype between hepatocytes and cholangiocytes was caused by chronic liver injury (Fig. 2a–d).

Compared with the WT-TAA group, atypical cholangiocytes determined by the colocalization of ALB/CK19 or ALB/SOX9 were significantly decreased in COX-2 knockout mice treated with TAA (KO-TAA, Fig. 2c–d). Consistently, oral administration of imrecoxib, a selective COX-2 inhibitor, also ameliorated atypical cholangiocytes in TAA-treated mice (Fig. 2e). Because COX-2 was involved in the development of HCT-derived atypical cholangiocytes, a mouse model of hepatocyte-specific COX-2 deletion was established. Atypical cholangiocyte proliferation was significantly reduced in this model (Fig. 2f), suggesting that the atypical cholangiocytes might be derived from chronically injured hepatocytes. Possibly, hepatocytes undergo hepatocyte-to-duct conversion in a COX-2-dependent manner.

### Biliary structure and bile drainage were impaired by HCT

**Fig. 3:**
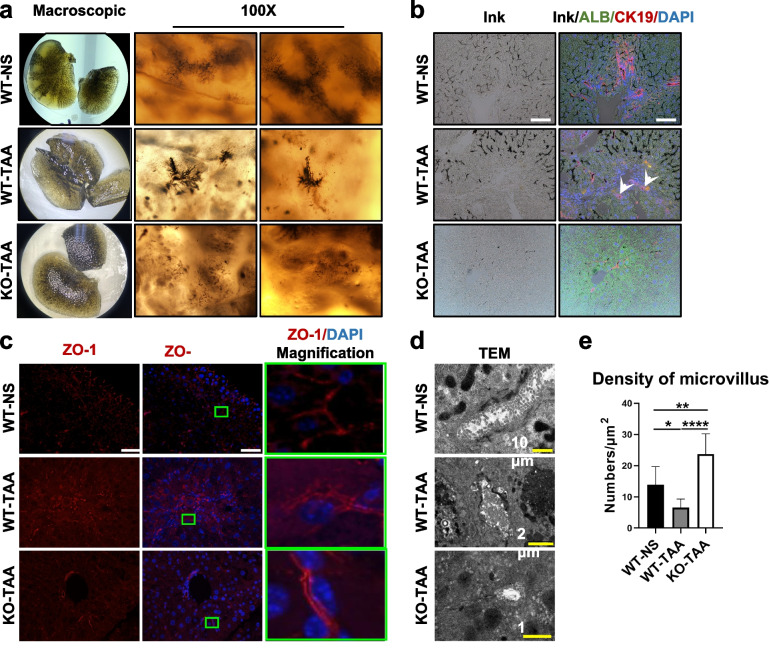
The structure of the biliary drainage system was impaired by HCT. **a** Visualization of the biliary tree structure in murine livers of the WT-NS, WT-TAA, and KO-TAA groups by retrograde ink injection and optical liver clearing. **b** HCT was detected in ink-injected murine liver of the three groups by IF of hepatocyte marker (ALB) and cholangiocyte markers (CK19). White arrows indicate coexpression or colocalization of hepatocyte and cholangiocyte markers. **c** Visualization of the bile canaliculi marked by IF of ZO-1 in the murine liver of the three groups. **d** The ultrastructure of the bile canaliculi shown by TEM in the three groups. **e** Quantification of the density of the microvilli in the bile canaliculi. White scale bars 50μm.*p < 0.05, **p < 0.01, ****p < 0.0001 (one-way ANOVA, *n* = 6/group)

The biliary tree was visualized by retrograde ink injection from the murine gallbladder and optical liver clearing. Compared to the regular biliary branches in the control group, the biliary branches were disorganized in the WT-TAA group, characterized by a tangled biliary plexus (Fig. 3a). Furthermore, the dysfunctional bile ducts were visualized by IF staining of CK19 and ALB in liver sections injected with ink. Most of the bile ducts were filled with ink in the control group, while the ink was absent in some HCT-derived bile ducts of the WT-TAA group (Fig. 3b, white arrows). The disorganized biliary branches and dysfunctional bile ducts could be partially attenuated by COX-2 knockout (Fig. 3a, b).

Bile canaliculi are the origin of the biliary system and are surrounded by the apical membranes of hepatocytes. They presented as a regular “train-track”-like structure by IF staining of ZO-1 in the WT-NS group. After treatment with TAA (WT-TAA group), the bile canaliculus became dilated and distorted. Figure 3c shows that the TAA-induced insult to the bile canaliculus was relieved by the deletion of COX-2 (KO-TAA group). Furthermore, the ultrastructure of the bile canaliculus by TEM revealed that the lumen of the bile canaliculus was obstructed by dense deposits in the WT-TAA group, which was absent in the WT-NS and KO-TAA groups (Fig. 3d). As a result, HCT not only disrupted the structure of the biliary system but also affected bile drainage.

### HCT dysregulated bile acid metabolism via abnormal bile acid transportation

**Fig. 4:**
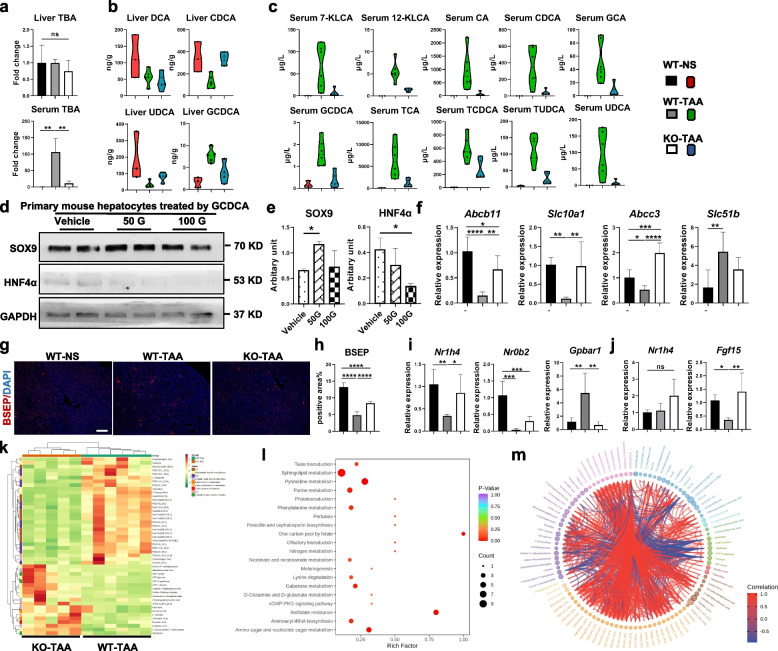
HCT-affected bile acid homeostasis through intervention in bile acid transportation. **a** The total bile acid (TBA) level in the liver and serum in the WT-NS, WT-TAA, and KO-TAA groups. **b** and **c** The concentrations of bile acid species in liver tissue (**b**) and serum (**c**) in the three groups were measured, with most changes presented as violin plots. **d** and **e** The effects of GCDCA on SOX9 and HNF4α protein levels in hepatocytes isolated from wild-type mice. **f** mRNA expression of bile acid transporters in murine livers of the three groups (qPCR). **g** and **h** Hepatic expression of BSEP in the three groups (IF) and its quantification. **i** and **j** mRNA levels of bile acid receptors in the liver tissue (**i**) and the ileum (**j**) of the three groups (qPCR). **k**–**m** Metabolomic analysis of liver tissues of the WT-TAA and KO-TAA groups. The heatmap of significantly altered metabolites (**k**), the bubble chart of enriched KEGG pathways (**l**), and the chord diagram showing the cross-linking of differential metabolites (**m**) are presented Images were cropped and scaled for illustration purposes from original Western blot images provided in supplementary files. Scale bars 50μm. *p < 0.05, **p < 0.01, ***p < 0.001, ****p < 0.0001; ns, not significant (one-way ANOVA, *n* = 6/group)

BA circulation is regulated by bile drainage. Therefore, it is necessary to assess whether abnormal bile drainage due to HCT disturbs BA homeostasis in the liver and serum. The data showed that the total serum BA of mice in the WT-TAA group was approximately 100 times higher than that in the WT-NS group and the KO-TAA group (Fig. 4a). However, the total hepatic BA levels were comparable among the three groups (Fig. 4a). Compared to the WT-NS group, hepatic chenodeoxycholic acid and ursodeoxycholic acid were significantly decreased, but 13 kinds of BAs in serum increased greatly in the WT-TAA group. Such changes tended to be restored to normal levels in the KO-TAA group (Fig. 4b, c). Hepatic glycochenodeoxycholic acid (GCDCA) was significantly elevated in the mice of the WT-TAA group compared to the WT-NS group. Then, primary mouse hepatocytes were incubated with GCDCA in vitro. The enhancement of SOX9 and the decrease in HNF4α in hepatocytes in vitro indicated that HCT could be induced directly by GCDCA (Fig. 4d, e). Hence, a higher level of GCDCA in vivo might promote HCT.

The mRNA levels of BA transporters, including the basolateral BA transporter Na^+^-taurocholate cotransporting polypeptide (NTCP, *Slc10a1*) and the apical bile salt export pump (BSEP, *Abcb11*), were decreased in the WT-TAA group compared to the WT-NS group. However, these decreases were abrogated in the KO-TAA group (Fig. 4f). The reduced protein level of BSEP induced by TAA was also recovered in the KO-TAA group (Fig. 4g, h). In addition, BSEP is expressed on the microvilli of the bile canaliculi, and the density of microvilli was significantly decreased in the WT-TAA group and improved in the KO-TAA group (Fig. 3e). Moreover, the mRNA level of basolateral BA efflux transporter Ostβ (*Slc51b*) in the WT-TAA group increased compared with the control group and decreased after COX-2 ablation (Fig. 4f). However, the mRNA expression of the major BA synthesis enzyme *Cyp7a1* in the liver was similar among the three groups (Supplementary Fig. S1). These data indicated that the alteration of the BA pool was mainly associated with disruption of BA transportation but not BA synthesis.

Activation of BA receptors, such as farnesoid X receptor (FXR) and Takeda G protein-coupled receptor 5 (TGR5), can regulate BA synthesis and BA transportation. The mRNA levels of FXR (*Nr1h4*) and its downstream molecule SHP-1 (*Nr0b2*) in the liver were significantly decreased in the WT-TAA group and increased in the KO-TAA group (Fig. 4i). In contrast, the expression of TGR5 (*Gpbar1*) was increased in the WT-TAA group and reduced after COX-2 deletion (Fig. 4i). Notably, FXR and the FXR downstream factor fibroblast growth factor 15 (FGF15) in the ileum also participate in bile acid signaling. The mRNA levels of *Fgf15* in the ileum were significantly decreased in the WT-TAA group and increased after COX-2 knockout (Fig. 4j). Moreover, accumulation of glycerolipids and phosphoglycerides was observed in the WT-TAA group (Supplementary Fig. S2a–c). COX-2 ablation partly redressed the metabolic disturbance after TAA-induced chronic liver injury (Fig. 4k–m). In brief, HCT resulted in BA metabolism dysregulation via abnormal BA transportation, while COX-2 was involved in this series of events.

### HCT was mediated by the TGF-β/β-catenin signaling pathway

**Fig. 5:**
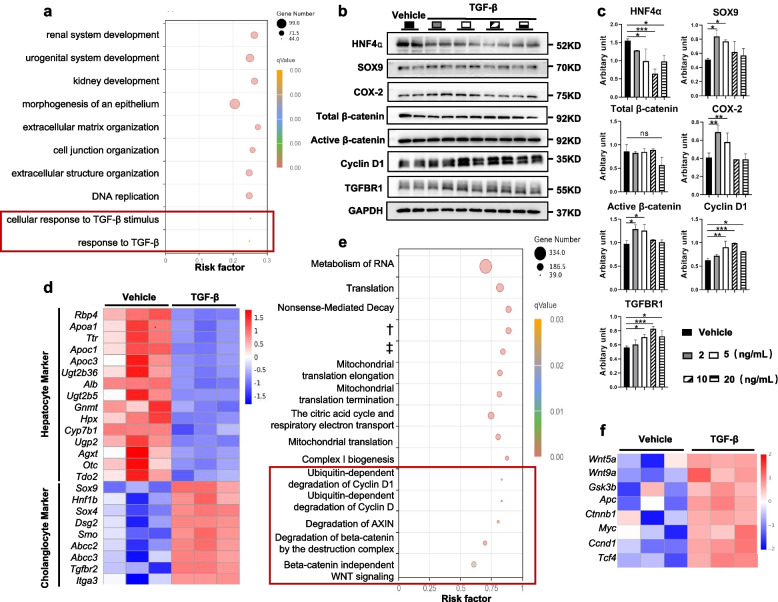
The TGF-β-TGFBR1-β-catenin signaling pathway was involved in HCT. **a** RNA sequencing of the livers of the WT-TAA and KO-TAA groups. The enrichment pathways of downregulated differentially expressed genes (KO-TAA group vs. WT-TAA group) are presented as a bubble chart. **b** and **c** Primary mouse hepatocytes were isolated and treated with different concentrations of TGF-β for 24 h. The protein levels of HNF4α, SOX9, COX-2, total and active β-catenin, Cyclin D1, and TGFBR1 were measured by WB. **d** RNA sequencing of primary mouse hepatocytes treated with vehicle or TGF-β. The expression of hepatocyte and cholangiocyte markers is presented as a heatmap. **e** The enrichment pathways of upregulated differentially expressed genes (TGF-β vs. vehicle) are presented as a bubble chart. **f** The mRNA expression of critical molecules in the β-catenin pathway is presented as a heatmap. Images were cropped and scaled for illustration purposes from original Western blot images provided in supplementary files. †Nonsense-Mediated Decay enhanced by the Exon Junction Complex. ‡Respiratory electron transport, ATP synthesis by chemiosmotic coupling, and heat production by uncoupling proteins. *p < 0.05, **p < 0.01, ***p < 0.001; ns, not significant (one-way ANOVA, *n* = 3/group)

To understand the underlying mechanisms of COX-2-mediated HCT during chronic liver injury, enrichment analysis of RNA-sequencing data from liver tissues of the KO-TAA and WT-TAA groups was performed. The TGF-β signaling pathway was downregulated in the KO-TAA group and was thus investigated in this study (Fig. 5a). HCT could be induced by TGF-β in primary mouse hepatocytes in vitro, accompanied by upregulation of TGF-β receptor 1 (TGFBR1, Fig. 5b, c). As shown by RNA sequencing, TGF-β treatment in primary mouse hepatocytes resulted in elevated expression of cholangiocyte markers (*Sox9*, *Hnf1β*, *Smo*, *Itga3*, etc.) and decreased expression of hepatocyte markers (*Alb*, *Ttr*, *Tdo2*, etc.) (Fig. 5d).

In the KEGG pathway analysis of differentially expressed genes between vehicle- and TGF-β-treated primary mouse hepatocytes, Wnt-β-catenin-related signaling pathways were significantly enriched, such as degradation of beta-catenin by the destruction complex, ubiquitin-dependent degradation of Cyclin D1, and degradation of AXIN (Fig. 5e). Consistently, the mRNA levels of key molecules in the Wnt-β-catenin signaling pathway, such as *Wnt5a*, *Wnt9a*, *Gsk3b*, *Apc*, *Ctnnb1*, *Ccnd1*, *Myc*, and *Tcf4*, and the protein expression of active β-catenin and Cyclin D1 were significantly upregulated in TGF-β-treated primary mouse hepatocytes compared with vehicle-treated cells (Fig. 5b, c, f). Moreover, TNFα and osteopontin were reported to induce HCT in vitro, and AML-12 cells treated with TNFα and osteopontin showed upregulation of the β-catenin pathway (Supplementary Fig. S3a–d).

### COX-2 inhibition ameliorated HCT by downregulating the TGF-β/β-catenin signaling pathway

**Fig. 6:**
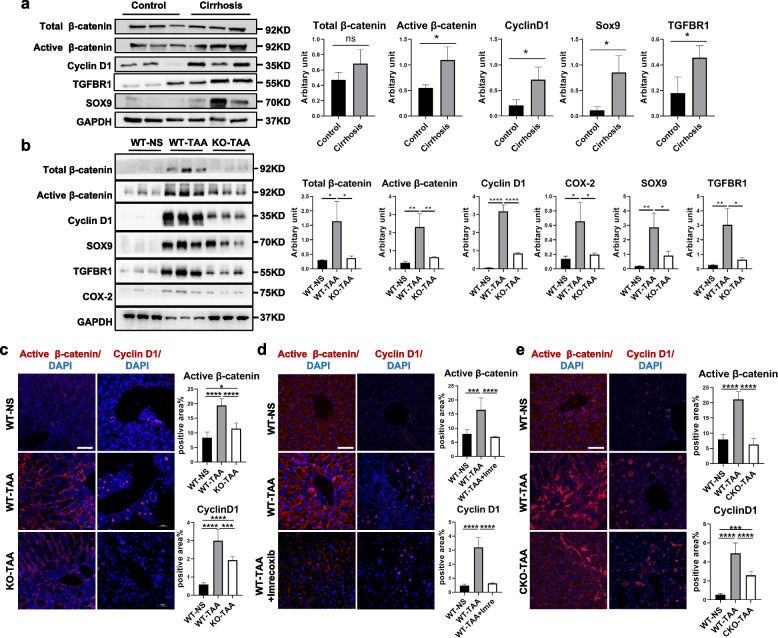
COX-2 inhibition downregulated the TGF-β-TGFBR1-β-catenin signaling pathway in vivo. **a** Comparison of the key protein levels of the TGF-β-TGFBR1-β-catenin signaling pathway in liver tissues between cirrhotic patients and healthy controls (WB). **b** Comparison of the key protein levels of the TGF-β-TGFBR1-β-catenin signaling pathway and COX-2 in the livers of the WT-NS, WT-TAA, and KO-TAA groups (WB). **c** The effects of panknockout of COX-2 on the expression of active β-catenin and Cyclin D1 in liver sections (IF). **d** The effects of pharmaceutical inhibition of COX-2 by imrecoxib on the expression of active β-catenin and Cyclin D1 in liver sections (IF). **e** The effects of hepatocyte-specific knockout of COX-2 on the expression of active β-catenin and Cyclin D1 in liver sections (IF). Images were cropped and scaled for illustration purposes from original Western blot images provided in supplementary files. Scale bars 50μm. *p < 0.05, **p < 0.01, ***p < 0.001, ****p < 0.0001; ns, not significant (one-way ANOVA, *n* = 3/group)

**Fig. 7:**
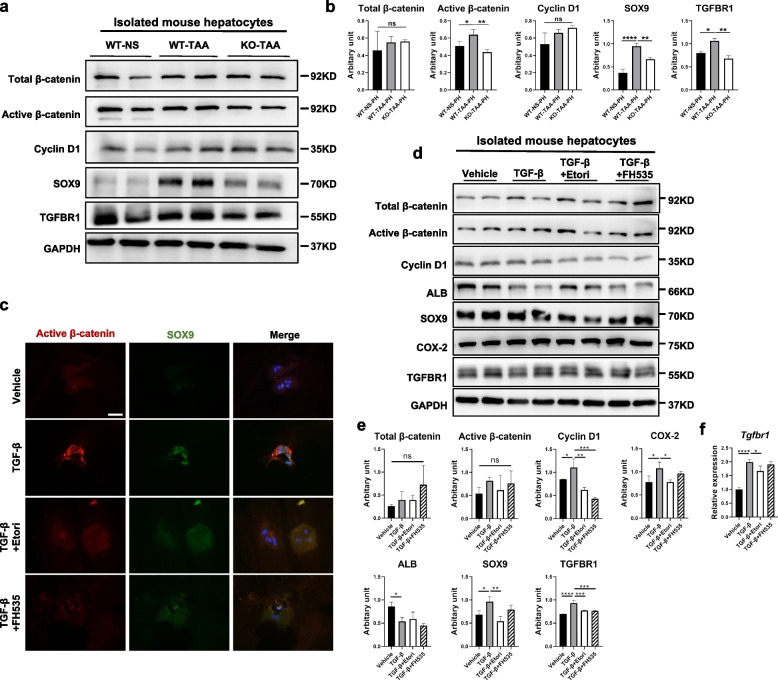
COX-2 inhibition ameliorated HCT by inhibiting the TGF-β-TGFBR1-β-catenin signaling pathway in vitro. **a** and **b** Comparison of the key protein levels of the TGF-β-TGFBR1-β-catenin signaling pathway in isolated murine hepatocytes among the WT-NS, WT-TAA, and KO-TAA groups (WB). **c** Primary mouse hepatocytes were isolated from wild-type mice and treated with vehicle, TGF-β, etoricoxib (COX-2 inhibitor), and FH535 (β-catenin pathway inhibitor). Active β-catenin and SOX9 were costained in these treated cells (IF). **d** and **e** Quantification of the key protein levels of the TGF-β-TGFBR1-β-catenin signaling pathway and COX-2 after treatments in hepatocytes isolated from wild-type mice (WB). **f** Measurement of the mRNA expression of *Tgfbr1* after treatment (qPCR). Images were cropped and scaled for illustration purposes from original Western blot images provided in supplementary files. Scale bars 50μm. *p < 0.05, **p < 0.01, ***p < 0.001, ****p < 0.0001; ns, not significant (one-way ANOVA, *n* = 3/group)

Next, whether COX-2 inhibition can attenuate HCT by regulating the TGF-β-TGFBR1-β-catenin signaling pathway was validated in vivo and in vitro. In vivo, TGFBR1, active β-catenin, Cyclin D1, and SOX9 were significantly increased in TAA-induced cirrhotic murine liver and cirrhotic human liver (Fig. 6a, b). However, the expression of these proteins was significantly lower in the livers of the KO-TAA group than in the WT-TAA group, as shown by Western blotting (Fig. 6b). In the mouse model, IF revealed that increased active β-catenin and Cyclin D1 expression after TAA administration was ameliorated by COX-2 deletion (Fig. 6c). Consistently, administration of imrecoxib in vivo (Fig. 6d) and hepatocyte-specific COX-2 knockout (Fig. 6e) decreased the active β-catenin and Cyclin D1 induced by TAA. Furthermore, primary mouse hepatocytes isolated from the WT-NS, WT-TAA, and KO-TAA groups showed that active β-catenin, Cyclin D1, and SOX9 were elevated in the WT-TAA group compared to those in the WT-NS group, while these increased proteins induced by TAA were significantly reduced in the primary mouse hepatocytes isolated from the KO-TAA group (Fig. 7a, b).

Next, we validated the effect of COX-2 inhibition on HCT in vitro. The increased HCT induced by TGF-β in primary mouse hepatocytes was significantly abrogated by the selective COX-2 inhibitor etoricoxib (Fig. 7c–e). TGF-β-induced HCT was accompanied by an increased protein level of TGFBR1, which was also abrogated by COX-2 inhibitors (Fig. 7d–e). Additionally, the β-catenin signaling pathway was also activated by TGF-β treatment, as determined by increased protein levels of active β-catenin, CyclinD1, and SOX9 in primary mouse hepatocytes upon TGF-β treatment (Fig. 7c–e). However, the enhanced protein levels of active β-catenin, CyclinD1, and SOX9 in primary mouse hepatocytes upon TGF-β stimulation were suppressed by etoricoxib (Fig. 7c–e). The β-catenin inhibitor FH535 also significantly reduced the activation of the β-catenin signaling pathway and ameliorated TGF-β-induced HCT (Fig. 7c–e). Confirmingly, etoricoxib and FH535 also ameliorated TNFα-induced HCT in primary mouse hepatocytes (Supplementary Fig. S4). Notably, the mRNA expression of *Tgfbr1* decreased after COX-2 inhibition but remained unchanged after β-catenin pathway inhibition (Fig. 7f), indicating that TGF-β/TGFBR1 might be upstream of the β-catenin signaling pathway.

**Fig. 8:**
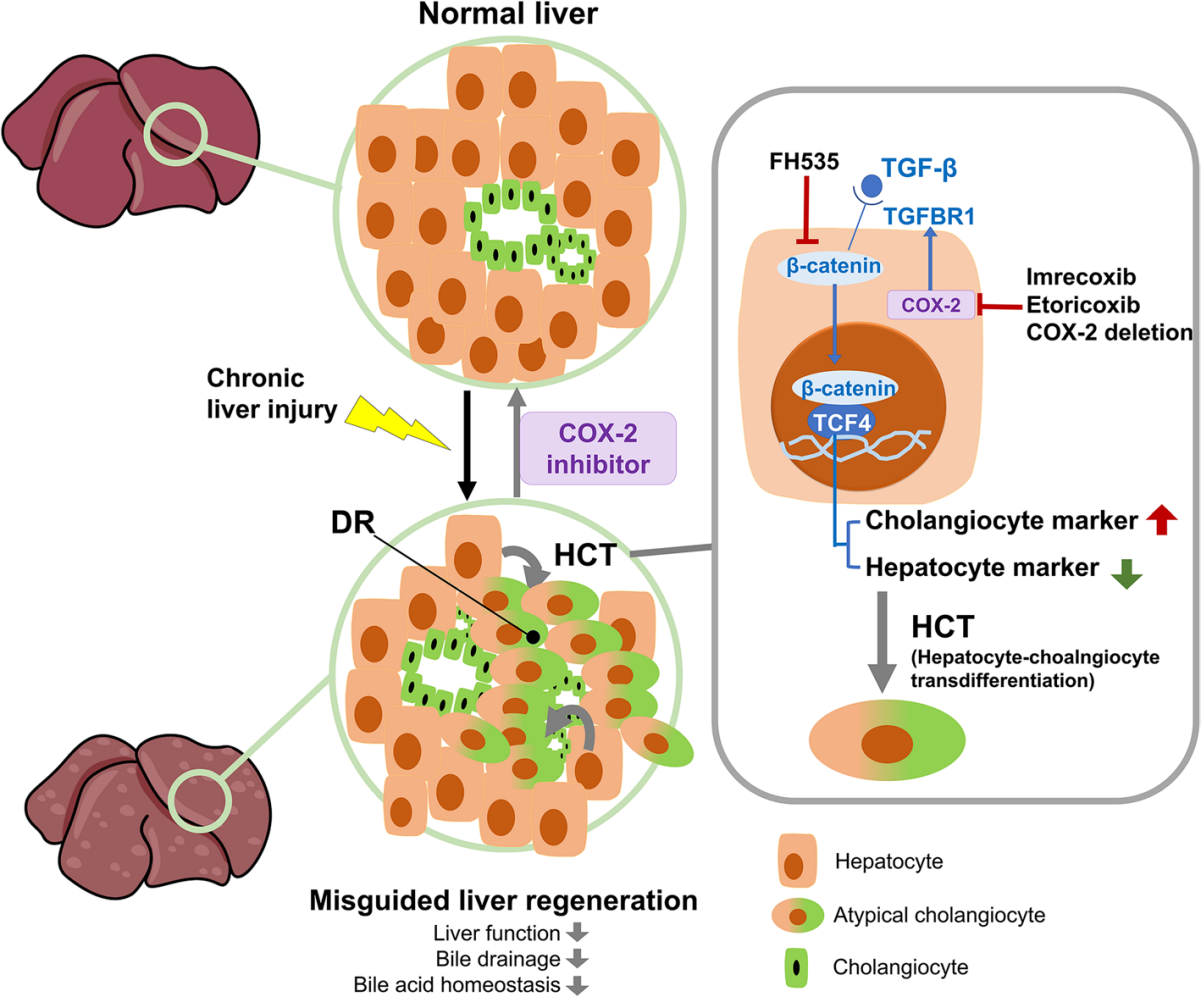
Schematic representation. Atypical cholangiocytes in ductular reaction (DR) can be derived from hepatocyte-cholangiocyte transdifferentiation (HCT). HCT mediated by the COX-2-TGF-β-TGFBR1-β-catenin-TCF4 pathway misguided liver regeneration with maldevelopment of the bile drainage system and bile acid homeostasis disequilibrium. The secondary strike of chronic liver injury can be ameliorated by the inhibition of COX-2

Activated β-catenin can enter the nucleus and bind the transcription factor transcription factor 4 (TCF4) to induce the transcription of target genes [20]. In the JASPAR database, markers of hepatocytes and cholangiocytes, such as *Sox9*, *Ck19*, *Alb*, and *Hnf4a*, were predicted as potential targets of TCF4 (Supplementary Fig. S5). These data indicated that COX-2 inhibition ameliorated HCT by downregulating the TGF-β-TGFBR1-β-catenin-TCF4 signaling pathway (Fig. 8).

## Discussion

Hepatocytes and cholangiocytes, the two mature hepatic epithelial cells, are derived from the hepatoblasts of the embryonic liver in the process of embryogenesis. During postnatal life, liver regeneration in the situation of compensated injury is mediated by hepatocyte mitosis [21]. However, chronic liver injury may cause cirrhosis by impairing liver regeneration and function. This study detected intrahepatic DR as atypical cholangiocytes in the liver tissue sections of patients with HBV cirrhosis as well as mice treated with TAA, BDL, and a 0.1% DDC diet, indicating the universality of DR in chronic hepatic injury.

Regarding the origin of cholangiocytes in DR, experimental studies with rodent models have demonstrated that oval cells, which are derived from HPCs with properties of biliary epithelium, are potential origins [22]. The precursors of oval cells may be the cells residing in the canals of Hering. Nevertheless, there has been a long-standing debate on whether progenitors are derived from biliary-like stem cells that acquire hepatocyte functions or from hepatocytes that lose hepatocyte functions [23]. The atypical cholangiocytes with intermediate genetic phenotypes in this study might be bipotential. It is worth noting that their proliferation is not only affected by various chronic liver injuries but also mediated by COX-2. Hepatocyte-specific COX-2 deletion significantly reduced atypical DR, suggesting that hepatocytes undergo HCT. Such a hepatocyte-to-duct conversion was also confirmed in our study in vitro.

Generally, bipotential HPCs can regenerate both hepatocytes and cholangiocytes [21, 24]. Hepatocytes may act as facultative liver stem cells and can replenish the cholangiocyte population after severe biliary injury [4]. Tarlow et al. considered that hepatocytes can escape injury through ductal metaplasia and redifferentiate into functional hepatocytes after damage subsides [24]. Therefore, HCT has been deemed a rescue for hepatocytes or biliary damage. Unless the cells become malignant, the benefits of DR are usually positive. However, whether HCT-origin cholangiocytes can obtain normal biliary function remains unclear. This study tried to address this question in terms of biliary structure and function. The HCT-derived bile ducts in this study were not marked with ink in the liver sections by retrograde ink injection, while most of the bile ducts were filled with ink in the control group, suggesting that HCT-derived cholangiocytes were not able to coalesce with the preexisting bile duct. The biliary branches were disorganized, and the tangled biliary plexus was present in the livers with chronic injury but without cirrhotic pseudolobules. Moreover, DR also affected the bile canaliculus, the origin of the biliary system surrounded by apical membranes of hepatocytes. They were dilated, distorted, and obstructed in contrast with those in the control group. These morphological changes in the whole duct may reduce the effectiveness of biliary drainage. In addition to the changes in biliary structure, HCT-derived DR also dysregulated BA metabolism via abnormal BA transportation, including decreased BSEP and NTCP. If the loss of normal hepatocytes is in exchange for immature cholangiocytes with deteriorated function, HCT would not be beneficial in liver regeneration but a second strike on hepatocytes. The data of this study showed that COX-2 is involved in several targets of the HCT process. It may be possible that HCT through other pathways would be beneficial for liver regeneration, but HCT regulated by COX-2 may exacerbate chronic liver injury by abrogating biliary homeostasis.

As we verified that COX-2 dominated the HCT process in chronic liver injury, the regulation of COX-2 on β-catenin pathway-mediated HCT remained elusive. Arachidonic acid (AA) can be catalyzed by COX-2 and converted to PGE2. The PGE2-β-catenin interaction plays an important role in liver regeneration after partial hepatectomy [25]. However, there is less concern regarding the link between COX-2 and the β-catenin pathway in chronic hepatic injury. Enrichment analysis of RNA-sequencing data from liver tissues of the KO-TAA and WT-TAA groups provided useful clues for us to reveal the mechanisms behind COX-2-mediated HCT. COX-2 deletion was related to downregulated TGF-β signaling pathways. It has been reported that the TGF-β signaling pathway is critical to liver homeostasis and regeneration. TGF-β can modulate biliary differentiation during liver development. Activation of TGFBR1 is capable of triggering HCT in the liver of mouse models with aberrant biliary development [26, 27]. This study showed that TGF-β-TGFBR1 pathway-induced upregulation of the β-catenin pathway and HCT could be ameliorated by inhibition of COX-2 in vitro. Further bioinformatics analysis enriched Wnt-β-catenin-related signaling pathways between vehicle- and TGF-β-treated primary mouse hepatocytes. Experiments with primary mouse hepatocytes showed that Wnt-β-catenin was significantly upregulated by TGF-β treatment. The Wnt/β-catenin pathway is a vital signaling pathway that drives liver development and regeneration and maintains liver homeostasis. Wnt/β-catenin also regulates cholangiocyte proliferation and determines the fate of bipotential liver progenitor cells [28–30]. In the context of HCT, Wnt proteins can induce HCT in AML-12 and primary mouse hepatocytes through activation and nuclear translocation of β-catenin [29]. Overexpression of hepatic β-catenin may prompt HCT in DDC-treated mice [19]. Thus, we demonstrated that inhibition of COX-2 downregulates HCT through the TGF-β/β-catenin signaling pathway and reverses the detrimental effect caused by HCT.

In summary, atypical cholangiocytes in DR derived from HCT mediated by the COX-2-TGF-β-TGFBR1-β-catenin pathway misguided liver regeneration with maldevelopment of the bile drainage system and bile acid homeostasis disequilibrium. The secondary strike of chronic liver injury can be ameliorated by the inhibition of COX-2.

## Supplementary Information

**Additional file 1: Supplementary methods. Supplementary Fig S1.** The mRNA expression of liver bile acid synthesis enzymes after TAA administration and COX-2 knockout; **Supplementary Fig S2.** Liver metabolomic changes between the control group and TAA-induced chronic liver injury group. **Supplementary Fig S3.** OPN and TNFα induced HCT and upregulation of the β-catenin pathway in AML-12 cells. **Supplementary Fig S4.** Inhibition of COX-2 and the β-catenin signaling pathway ameliorated TNFα-induced HCT; **Supplementary Fig. S5.** Prediction of potential targets of TCF4 using the JASPAR database. **Supplementary Table S1.** Clinical characteristics of patients with liver cirrhosis and normal controls. **Supplementary Table S2.** Antibodies used in this study. **Supplementary Table S3.** qPCR primers. 

**Additional file 2. **Original WB data.

## Data Availability

The datasets generated during and/or analyzed during the current study are available from the corresponding author on reasonable request.

## Abbreviations

DR: Ductular reaction
HPC: Hepatic progenitor cell
HCT: Hepatocyte-cholangiocyte transdifferentiation
HBV: Hepatitis B virus
NASH: Nonalcoholic steatohepatitis
ALD: Alcoholic liver disease
BA: Bile acid
COX-2: Cyclooxygenase-2
TGF-β: Transforming growth factor beta
TAA: Thioacetamide
DDC: 3,5-Diethoxycarbonyl-1,4-dihydrocollidine
BDL: Bile duct ligation
CK19: Cytokeratin 19
ALB: Albumin
HNF4α: Hepatocyte nuclear factor 4α
SOX9: Sex determining region Y-box 9
IF: Immunofluorescence
GCDCA: Glycochenodeoxycholic acid
NTCP: Na^+^-taurocholate cotransporting polypeptide
BSEP: The bile salt export pump
FXR: Farnesoid X receptor
TGF5: Takeda G protein-coupled receptor 5
TCF4: Transcription factor 4
