# Supplementary material for: Atypical cholangiocytes derived from hepatocyte-cholangiocyte transdifferentiation mediated by COX-2: a kind of misguided liver regeneration
Source: Inflamm Regen. 2023 Jul 14;43:37. doi: 10.1186/s41232-023-00284-4 (PMC10347763; doi:10.1186/s41232-023-00284-4)
Supplement: Supplementary file 1 — Additional file 1: Supplementary methods. Supplementary Fig S1. The mRNA expression of liver bile acid synthesis enzymes after TAA administration and COX-2 knockout; Supplementary Fig S2. Liver metabolomic changes between the control group and TAA-induced chronic liver injury group. Supplementary Fig S3. OPN and TNFα induced HCT and upregulation of the β-catenin pathway in AML-12 cells. Supplementary Fig S4. Inhibition of COX-2 and the β-catenin signaling pathway ameliorated TNFα-induced HCT; Supplementary Fig. S5. Prediction of potential targets of TCF4 using the JASPAR database. Supplementary Table S1. Clinical characteristics of patients with liver cirrhosis and normal controls. Supplementary Table S2. Antibodies used in this study. Supplementary Table S3. qPCR primers. [file 41232_2023_284_MOESM1_ESM.pdf]

Atypical cholangiocytes derived from hepatocyte-cholangiocyte transdifferentiation mediated by COX-2: a kind of misguided liver regeneration

Tian Lan[1,#], Yang Tai [1,2,#], Chong Zhao[1,2], Yang Xiao[1], Zhu Yang[1], Linhao Zhang[1,2], Can Gan[1,2], Wenting Dai[1,2], Huan Tong[1,2], Chengwei Tang[1,2], Zhiyin Huang[2,*], Jinhang Gao[1,*]

1. Laboratory of Gastroenterology and Hepatology, West China Hospital, Sichuan University, Chengdu 610041, China
2. Department of Gastroenterology, West China Hospital, Sichuan University, Chengdu 610041, China

[#] These authors contributed equally

***Corresponding author**

Jinhang Gao, Ph.D., Professor

Zhiyin Huang, M.D., Associated professor

Laboratory of Gastroenterology and Hepatology,

West China Hospital, Sichuan University

No. 37, Guo Xue Xiang

Chengdu, 610041, P. R. China

E-mail: Gao.jinhang@scu.edu.cn /huangzy911@163.com

Tel.: +86-28-8542383

Fax: +86-28-85582944

Supplementary methods

Isolation of mouse hepatocytes

Primary hepatocytes were isolated from mice in the WT-NS, WT-TAA, and KO-TAA groups using a previously described method.[1] In general, mice were anesthetized and the portal vein was cannulated. Then, the liver was perfused and digested with warm collagenase (Sigma-Aldrich, #C9891) solution. Digestion was stopped once cracks appeared on the surface of the liver. Next, the liver was excised and placed in a 10 cm disk containing cold isolation buffer. The liver was then torn apart with forceps and rinsed to release liver cells into the isolation buffer. The cell suspension was collected and filtered through a 70 μm pore size nylon strainer (Nest, #258368) and centrifuged at 150×g for 2 minutes. After centrifugation, the supernatant was discarded and the pellet was resuspended in 50% Percoll solution (Solarbio, #P8370), followed by density gradient centrifugation at 150×g for 10 minutes. Afterward, the pellets were washed with a culture medium and the viability of the yielded cells was measured by trypan blue staining. Cells were plated and cultured overnight before they became attached and showed a cuboidal shape and discernable nuclei.

Cell culture and treatment

The primary murine hepatocyte and the alpha mouse liver 12 (AML-12) cell line (Procell Life Science & Technology, #CL-0602) were cultured in DMEM: F12 medium (Gibco, #C11330500BT) containing 10% fetal bovine serum (FBS, Biological Industries, Cromwell, CT, USA), 1% penicillin/streptomycin (HyClone, #SV30010), 1%

insulin, transferrin, selenium solution (ITS-G, Thermo Fisher, #41400045), and dexamethasone (40 ng/mL, Sigma, #4902). The cells were incubated at 5% CO2 and 37°C. Cell line authentication was carried out by short tandem repeat profiling.

To induce HCT, AML-12 cells (serum-free starved for 2 hours first) and primary murine hepatocytes were treated with TGF-β (2-20 ng/mL, Peprotech, #100-21C), TNF-α (100 ng/mL, R&D, #210-TA), OPN (50-100 nM, R&D, #441-OP-050/CF), and GCDCA (50-100 μM, Selleck, #S5794) for 6-24 hours. To study the regulation of TGF-β-induced HCT, cells were pre-incubated with COX-2 inhibitor (Etoricoxib, 20 μM, MCE, #HY-15321) or β-catenin pathway inhibitor (FH535, 500 nM, MCE, #HY-15721) for 2 hours before TGF-β treatment. After treatment, cells were harvested for the following experiments.

Histology, immunohistochemistry (IHC), and immunofluorescence (IF)

Liver tissue was fixed in 4% paraformaldehyde and embedded in paraffin. Liver sections (4 μm) were stained with hematoxylin and eosin (H&E) following standard protocols. Detection of the cholangiocyte marker cytoskeleton keratin CK19 was performed. Liver sections were deparaffinized and rehydrated before antigen retrieval using sodium citrate buffer. Then, sections were incubated in 3% H_2O_2 for 15 minutes and blocked for 1 hour. After that, sections were incubated with primary antibodies overnight at 4 °C followed by incubation with horseradish peroxidase-conjugated secondary antibodies for 30 minutes. Finally, sections were stained with 3, 3′-diaminobenzidine (DAB) solution and counter-stained with hematoxylin.

For immunofluorescence, liver sections were deparaffinized, rehydrated, and antigen retrieved as described above. Cells, they were seeded on glass chamber slides and fixed with 4% paraformaldehyde for 15 minutes. The slides were then permeabilized with 0.5% Triton X-100 for 15 minutes and blocked for 1 hour. After incubation with primary antibodies, sections were incubated with fluorochrome-conjugated secondary antibodies in the dark for 1 hour. Finally, sections were coverslipped using antifade reagents with DAPI and visualized using a fluorescence microscope (Olympus, EX53) or a two-photon confocal microscope (Nikon A1R MP+). Three to five fields at 100× magnification were randomly selected for each section and semi-quantitative analysis. All images were analyzed by ImageJ software. All primary antibodies used are listed in Supplementary Table S2.

RNA extraction and quantitative real-time PCR

RNA was extracted from 20 mg of the frozen liver using a commercially available kit (Frogene, #RE-03011). 7 μL of RNA was reverse-transcribed using Revertaid first strand cDNA synthesis kit (Thermofisher, #K1622). Quantitative real-time PCR (qRT-PCR) was performed using SYBR Green Mix. The expression level of mRNA was determined by a CFX96 real-time PCR detection system (Bio-Rad) using the $2^{-\Delta\Delta Ct}$ method and shown as fold changes. Primer sequences are listed in Supplementary Table S3.

Transmission Electron Microscopy (TEM)

For transmission electron microscopy, the livers were perfused with normal saline, excised, and fixed in 2.5% glutaraldehyde (Solarbio, #P1126) overnight at 4℃. The samples were then washed with PBS and fixed with 1% osmium tetroxide solution before being dehydrated and embedded in araldite resin. Subsequently, 70-90 nm sections were stained with lead citrate and uranyl acetate and then imaged by TEM (H-600IV Hitachi, Tokyo, Japan).

Protein extraction and Western blot

Frozen liver tissues and cells were homogenized, and total proteins were extracted using a protein extraction kit (KeyGen Biotech, #KGBSP002). Equal amounts (50 μg for tissues and 30 μg for cells) of each sample were loaded onto an SDS-PAGE gel and separated before being transferred to PVDF membranes (Merck Millipore, #IPVH00010). Then, the membranes were blocked with 5% non-fat milk and incubated with primary antibodies overnight at 4 ℃. After incubation with horseradish peroxidase-conjugated secondary antibodies, protein bands were visualized using BeyoECL Star reagent (Beyotime, #P0018AM). The protein level was determined using ImageJ software and shown as relative expression to GAPDH. All primary antibodies used are listed in Supplementary Table S2.

Bile acid measurement

The concentrations of different bile acid species in liver tissue and serum were measured by Metware Biotechnology Inc (Wuhan, China). In general, 20 mg (± 1 mg)

of liver and 50 μL of serum collected from each group of mice were added to an internal standard and a steel ball, and then 200 μL of methanol was added to the homogenate. Samples were shaken at 2500 rpm for 10 minutes and then kept in a -20°C refrigerator for 10 minutes before being centrifuged at 12000 rpm for 10 minutes. After that, the supernatant was concentrated in the concentrator and reconstituted with 100 μL of 50% methanol-water for LC-MS/MS analysis. The sample extracts were analyzed using an LC-ESI-MS/MS system (Ultra Performance Liquid Chromatography, UPLC, Shim-pack UFLC SHIMADZU CBM30A, http://www.shimadzu.com.cn/; Tandem mass spectrometry, MS/MS, Applied Biosystems 6500 QTRAP, http://www.appliedbiosystems.com.cn/).

mRNA sequencing and data analysis

Mouse liver tissues and primary mouse hepatocytes were collected, and transcriptome sequencing was performed by Novogene Co., Ltd (Beijing, China). Generally, RNA was extracted from liver tissues and cells, and a library was obtained for transcriptome sequencing. After the library was qualified, the different libraries are pooling according to the effective concentration and the target amount of data off the machine, and then sequenced by the Illumina NovaSeq 6000. The FPKM of each gene was calculated based on the length of the gene and reads count mapped to this gene. Differential expression analysis of two conditions/groups (three biological replicates per condition) was performed using the DESeq2 R package (1.20.0). The resulting *p*-values were adjusted using Benjamini and Hochberg's approach for

controlling the false discovery rate. padj<=0.05 and |log2(fold change)| >= 1 were set as the thresholds for significantly differential expression. Gene Ontology (GO) enrichment analysis of differentially expressed genes was implemented by the clusterProfiler R package (3.8.1). GO terms with corrected *p*-values less than 0.05 were considered significantly enriched by differentially expressed genes. ClusterProfiler R package (3.8.1) was used to test the statistical enrichment of differential expression genes in KEGG pathways. Reactome pathways with corrected *p*-values less than 0.05 were considered significantly enriched by differentially expressed genes. The raw data of mRNA-sequencing have been deposited in the NCBI Gene Expression Omnibus.

Liver metabolomic assay

The liver metabolomic assay was performed by Metware Biotechnology Inc. Generally, samples were thawed on ice, and hydrophilic and hydrophobic compounds were extracted. The sample extracts were analyzed using an LC-ESI-MS/MS system (UPLC, ExionLC AD, https://sciex.com.cn/; MS, QTRAP® System, https://sciex.com/). Unsupervised principal component analysis (PCA) was performed by statistics function prcomp within R (www.r-project.org). The hierarchical cluster analysis (HCA) results of samples and metabolites were presented as heatmaps with dendrograms, while Pearson correlation coefficients (PCC) between samples were calculated by the cor function in R and presented as only heatmaps. Both HCA and PCC were carried out by R package ComplexHeatmap. Significantly regulated metabolites between groups were

determined by VIP >= 1 and absolute Log2FC (fold change) >= 1. VIP values were extracted from the OPLS-DA result, which also contains score plots and permutation plots, and were generated using the R package MetaboAnalystR. Identified metabolites were annotated using the KEGG compound database (http://www.kegg.jp/kegg/compound/), and annotated metabolites were then mapped to the KEGG Pathway database (http://www.kegg.jp/kegg/pathway.html).

Retrograde ink injection and optical liver clearing

Mice were anesthetized and subjected to laparotomy, and the common bile duct was ligated on the distal side. Carbon black ink was slowly infused into the gallbladder using a 36G needle under a stereomicroscope. The injection was stopped when black dots appeared on the surface of the liver. Then, the whole liver was excised and fixed in 4% paraformaldehyde for 1 day. Afterward, the liver was washed in PBS, soaked in the Tissue-Clearing Reagent CUBIC-L (TCI, T3740), and gently shaken at 37°C for 1 week for delipidation. Finally, the liver was soaked in Tissue-Clearing Reagent CUBIC-R+(M) (TCI, T3741) and gently shaken at 37°C for 1 week for refractive index matching. After the optical clearing process, the biliary tree was visualized and observed using a stereomicroscope. The liver was sectioned after ink injection and fixation, and IF was performed using the same protocol as above.

Public data mining

JASPAR is an open-access database of curated and non-redundant transcription factor

binding profiles (https://jaspar.genereg.net/). The JASPAR database was applied to predict the regulatory effect of TCF4 on the gene expression of hepatocyte and cholangiocyte makers. Two datasets were used (MA0830.1 and MA0830.2).

References

[1] He L, Sehrawat TS, Verma VK, Navarro-Corcuera A, Sidhu G, Mauer A, et al. XIAP Knockdown in Alcohol-Associated Liver Disease Models Exhibits Divergent in vitro and in vivo Phenotypes Owing to a Potential Zonal Inhibitory Role of SMAC. Frontiers in physiology 2021;12:664222.

Supplementary figures

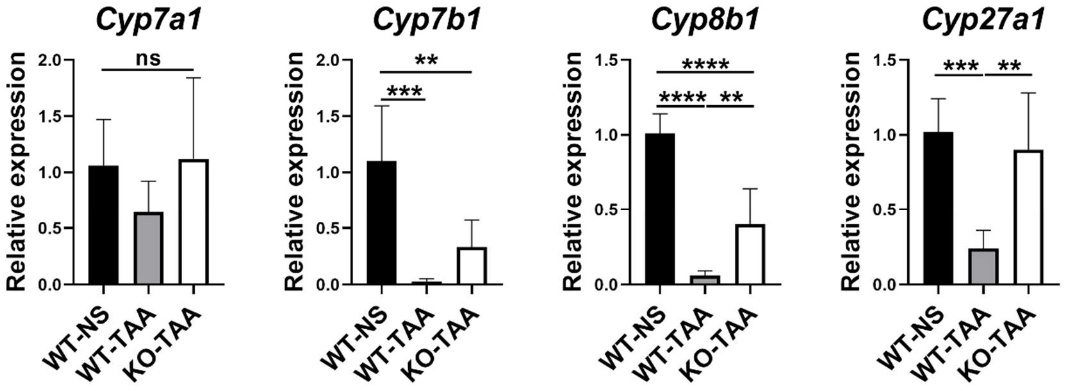

Supplementary Figure S1 The mRNA expression of liver bile acid synthesis enzymes after TAA administration and COX-2 knockout.

The expression of *Cyp7a1*, *Cyp7b1*, *Cyp8b1*, and *Cyp27a1* was measured in the livers of the WT-NS, WT-TAA, and KO-TAA groups by qPCR.

**p<0.01, ***p<0.001, **** p<0.0001; ns, not significant.

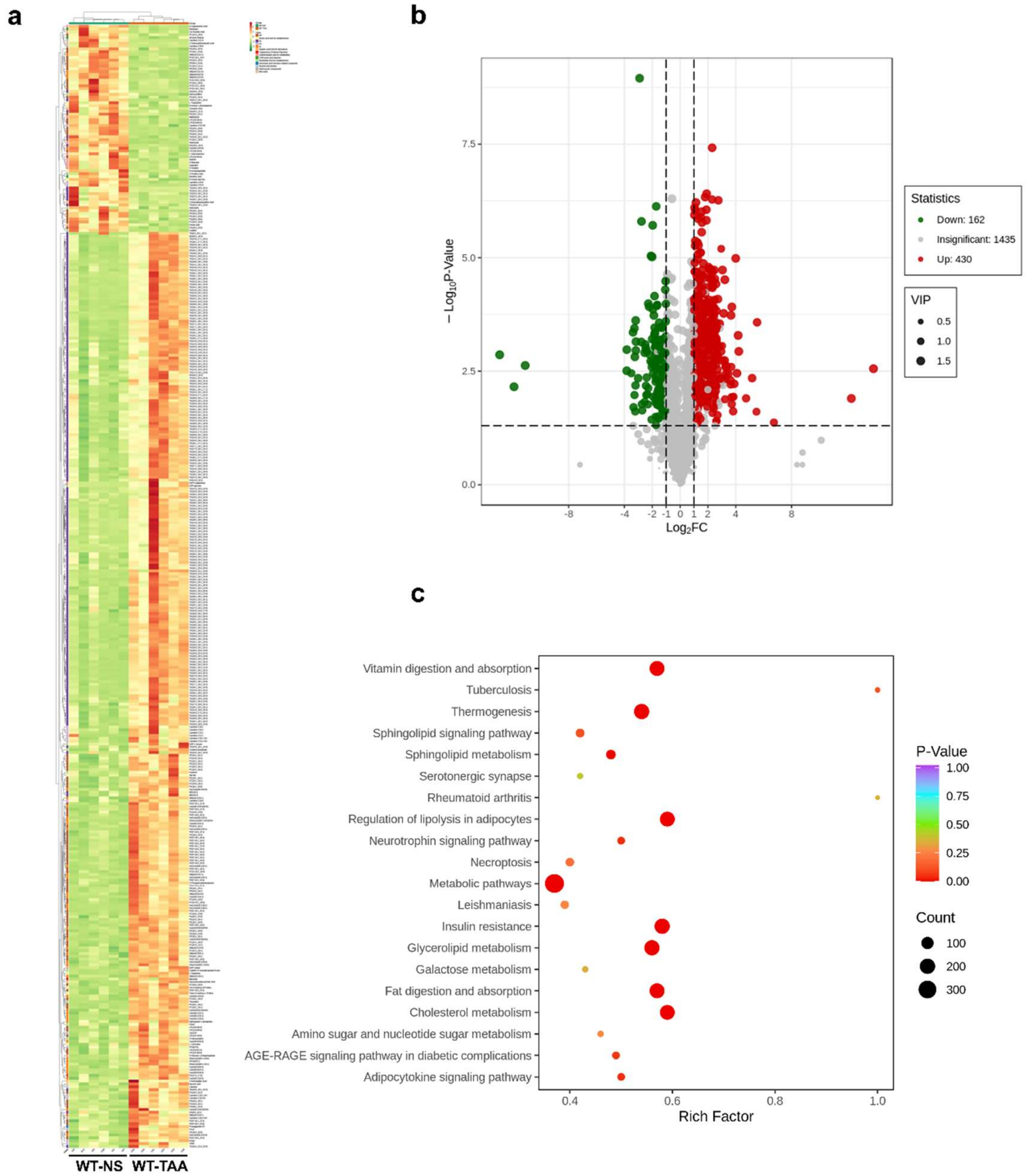

Supplementary Figure S2 Liver metabolomic changes between the control group and TAA-induced chronic liver injury group

a-c Metabolomic analysis was performed in the liver tissues of the WT-TAA and WT-NS groups. The heatmap of significantly altered metabolites (a), the volcano plot showing differential metabolites (b), and the bubble chart showing enriched KEGG pathways (c) are presented.

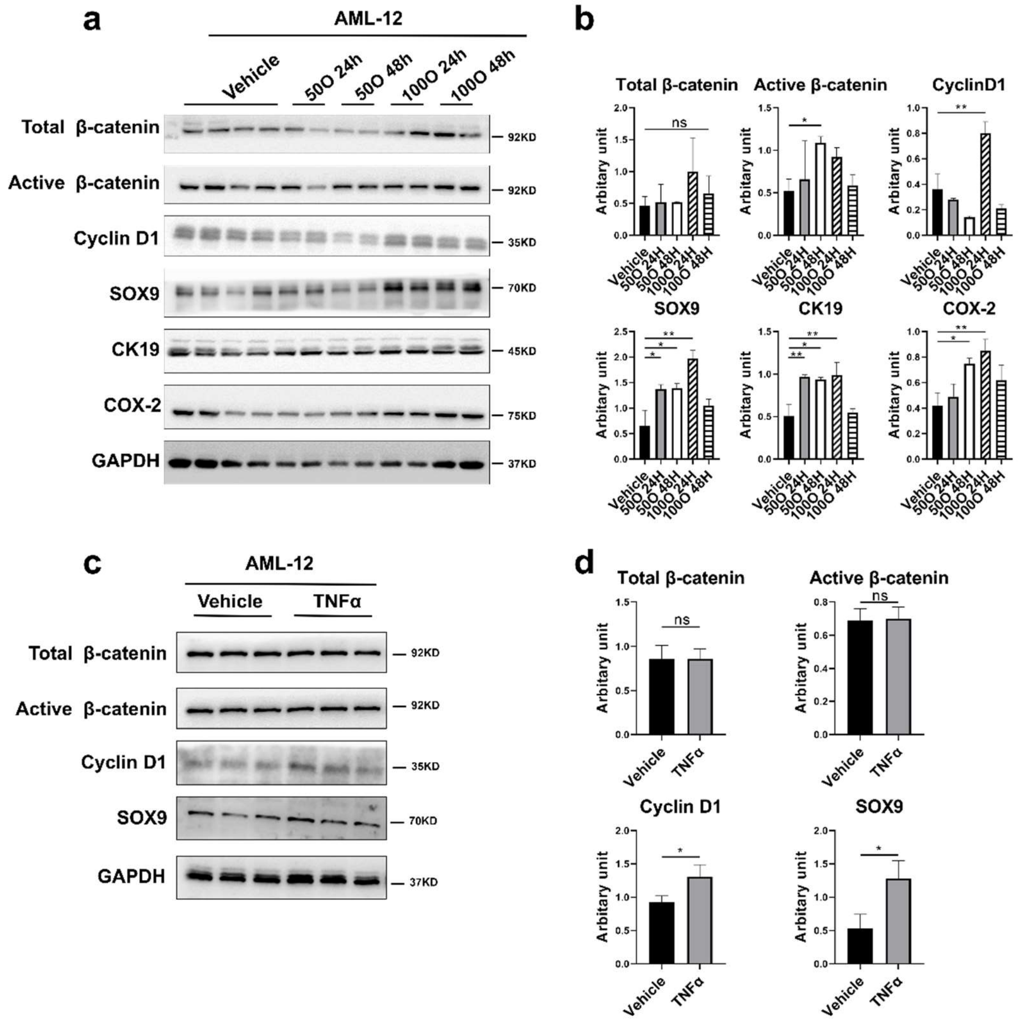

Supplementary Figure S3 OPN and TNFα induced HCT and upregulation of the β-catenin pathway in AML-12 cells

a,b AML-12 cells were treated with vehicle or different concentrations of OPN for 24 or 48 hours. The protein levels of total and active β-catenin, Cyclin D1, SOX9, CK19, and COX-2 were measured by WB.

c,d AML-12 cells were treated with vehicle or TNFα for 24 hours. The protein levels of total and active β-catenin, Cyclin D1, and SOX9 were measured by WB.

*p<0.05, **p<0.01; ns, not significant.

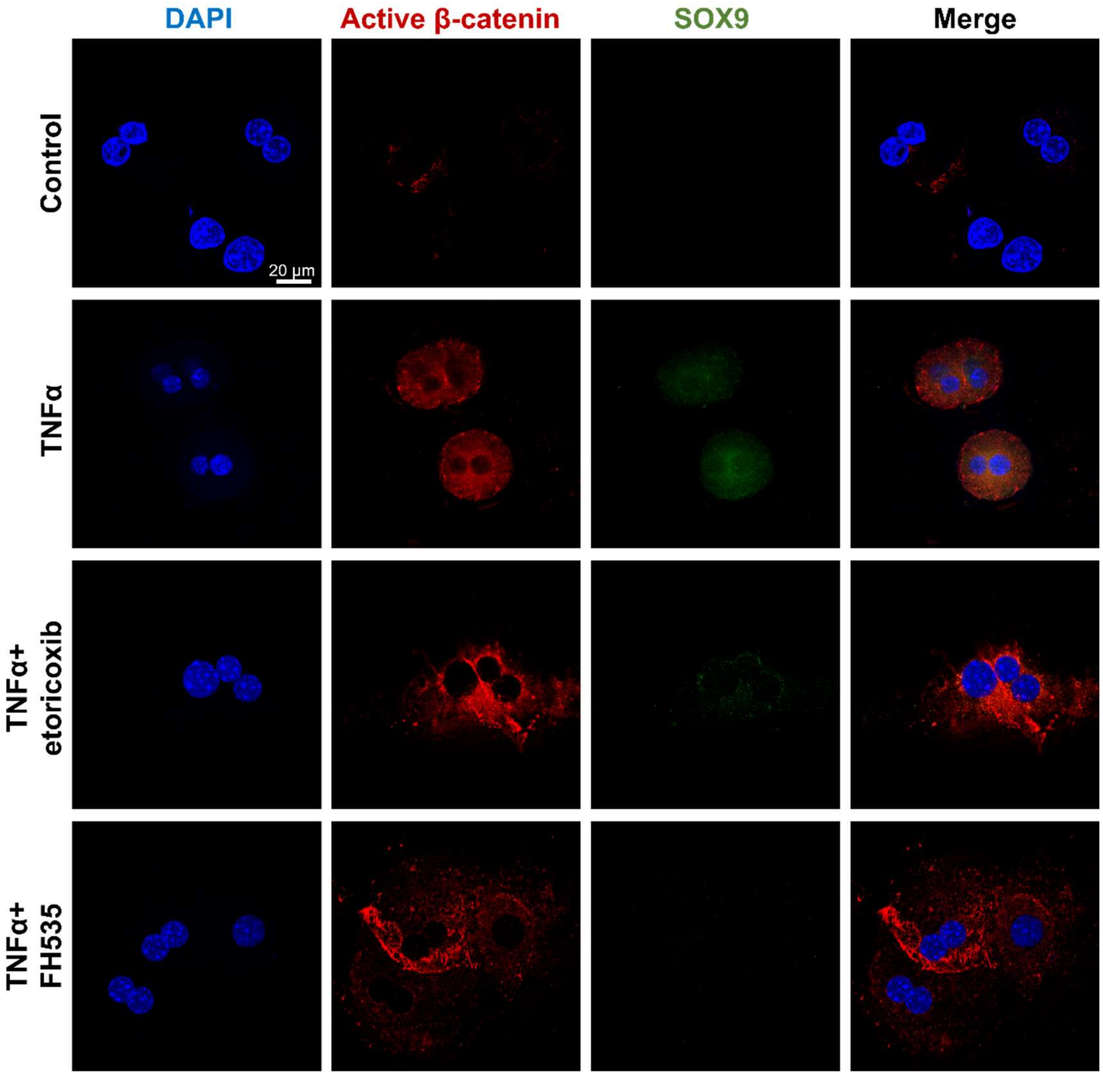

Supplementary Figure S4 Inhibition of COX-2 and the β-catenin signaling pathway ameliorated TNFα-induced HCT

Primary mouse hepatocytes were isolated from wild-type mice and treated with vehicle, TNFα, etoricoxib, and FH535. Active β-catenin and SOX9 were co-stained in these treated cells.

JASPAR: TCF4-SOX9

Matrix ID	Name	Score	Relative score	Sequence ID	Start	End	Strand	Predicted sequence
MA0830.2	MA0830.2.TCF4	9.821015	0.8703773866832557	NC_000017.11:72119920-72121019	479	491	+	CCCCACCAGCCCC
MA0830.1	MA0830.1.TCF4	8.609073	0.9190954214829148	NC_000017.11:72119920-72121019	12	21	-	AACACCTGAA
MA0830.2	MA0830.2.TCF4	7.9568193	0.8325991511845601	NC_000017.11:72119920-72121019	378	390	+	GGGCTCCTGCGTG
MA0830.2	MA0830.2.TCF4	7.918486	0.8318264597418494	NC_000017.11:72119920-72121019	10	22	-	AAACACCTGAAGG
MA0830.2	MA0830.2.TCF4	7.635889	0.8261001988368835	NC_000017.11:72119920-72121019	613	625	-	ACGCGCCTGGAGC
MA0830.2	MA0830.2.TCF4	7.5685987	0.8247366961795146	NC_000017.11:72119920-72121019	1041	1053	-	AGCCACCCGCCAA
MA0830.2	MA0830.2.TCF4	7.3293576	0.819888956197596	NC_000017.11:72119920-72121019	683	695	-	CGGCACCGGGGAC
MA0830.2	MA0830.2.TCF4	7.114526	0.8155358214469782	NC_000017.11:72119920-72121019	1002	1014	+	AATCAGCTGCCTG
MA0830.1	MA0830.1.TCF4	6.6826463	0.8896330608607548	NC_000017.11:72119920-72121019	480	489	+	CGCACCAGCC
MA0830.2	MA0830.2.TCF4	6.5908194	0.8049239642737832	NC_000017.11:72119920-72121019	719	731	-	CCCCACCTCCGAC

JASPAR: TCF4-ALB

Matrix ID	Name	Score	Relative score	Sequence ID	Start	End	Strand	Predicted sequence
MA0830.1	MA0830.1.TCF4	7.0184317	0.8947684920228142	NC_000004.12:73402287-73404286	1834	1843	-	ACCATCTGGA
MA0830.1	MA0830.1.TCF4	5.012199	0.864085590196937	NC_000004.12:73402287-73404286	1418	1427	+	TGCATCTGAG
MA0830.1	MA0830.1.TCF4	4.843954	0.861512488995895	NC_000004.12:73402287-73404286	415	424	-	TACATCTGAA
MA0830.1	MA0830.1.TCF4	4.6517677	0.8585732312678559	NC_000004.12:73402287-73404286	495	504	+	TTCATCTGTA
MA0830.1	MA0830.1.TCF4	4.2372513	0.8522337038216828	NC_000004.12:73402287-73404286	476	485	+	CACACTTGTT
MA0830.1	MA0830.1.TCF4	4.22236	0.8520059617399525	NC_000004.12:73402287-73404286	1566	1575	+	CACACTTGGA
MA0830.1	MA0830.1.TCF4	3.786536	0.8453405587040018	NC_000004.12:73402287-73404286	1333	1342	-	CCCACATGTG
MA0830.1	MA0830.1.TCF4	3.7498229	0.8447790759124086	NC_000004.12:73402287-73404286	1333	1342	+	CACATGTGGG
MA0830.1	MA0830.1.TCF4	3.193932	0.8362773988780737	NC_000004.12:73402287-73404286	1344	1353	+	CACAGCAGGA
MA0830.1	MA0830.1.TCF4	1.8466085	0.815671715890078	NC_000004.12:73402287-73404286	1430	1439	+	AACATCAGCT

JASPAR: TCF4-HNF4A

Matrix ID	Name	Score	Relative score	Sequence ID	Start	End	Strand	Predicted sequence
MA0830.1	MA0830.1.TCF4	12.859345	0.9840981995980338	NC_000020.11:44353699-44355698	249	258	-	AGCACCTGTA
MA0830.1	MA0830.1.TCF4	12.553732	0.9794242106922582	NC_000020.11:44353699-44355698	1797	1806	+	TACACCTGGC
MA0830.1	MA0830.1.TCF4	11.496144	0.9632496878642588	NC_000020.11:44353699-44355698	1158	1167	-	GACACCTGCC
MA0830.1	MA0830.1.TCF4	11.1458235	0.9578919549353356	NC_000020.11:44353699-44355698	1094	1103	-	ACCACCTGCT
MA0830.1	MA0830.1.TCF4	11.115113	0.957422279280279	NC_000020.11:44353699-44355698	351	360	+	TCCACCTGCC
MA0830.2	MA0830.2.TCF4	12.920078	0.9331736610904011	NC_000020.11:44353699-44355698	1156	1168	-	AGACACCTGCCAC
MA0830.2	MA0830.2.TCF4	12.915314	0.9330771169319492	NC_000020.11:44353699-44355698	350	362	+	ATCCACCTGCCTC
MA0830.2	MA0830.2.TCF4	12.213579	0.9188578783890211	NC_000020.11:44353699-44355698	1092	1104	-	CACCACCTGCTTG
MA0830.1	MA0830.1.TCF4	8.468206	0.9169410422104237	NC_000020.11:44353699-44355698	1990	1999	+	GGCAGCTGGC
MA0830.1	MA0830.1.TCF4	7.8145866	0.9069447188855946	NC_000020.11:44353699-44355698	815	824	+	CCCATCTGTA

JASPAR: TCF4-CK19

Matrix ID	Name	Score	Relative score	Sequence ID	Start	End	Strand	Predicted sequence
MA0830.1	MA0830.1.TCF4	9.893434	0.9387381669163501	NC_000017.11:c41530308-41528309	991	1000	+	TGCAGCTGTT
MA0830.1	MA0830.1.TCF4	9.662196	0.9352016705471317	NC_000017.11:c41530308-41528309	991	1000	-	AACAGCTGCA
MA0830.1	MA0830.1.TCF4	9.0837755	0.926355426868055	NC_000017.11:c41530308-41528309	1982	1991	-	CTCACCTGGC
MA0830.1	MA0830.1.TCF4	9.043636	0.9257415464057956	NC_000017.11:c41530308-41528309	757	766	-	AGCATCTGCC
MA0830.1	MA0830.1.TCF4	8.290111	0.9142172822093413	NC_000017.11:c41530308-41528309	264	273	-	ATCACCTGGG
MA0830.1	MA0830.1.TCF4	7.1486206	0.8967595743375382	NC_000017.11:c41530308-41528309	1015	1024	+	GTCACCTGGG
MA0830.1	MA0830.1.TCF4	7.143394	0.8966796396307641	NC_000017.11:c41530308-41528309	255	264	-	GTCACCTGTG
MA0830.2	MA0830.2.TCF4	10.533874	0.8848220237578459	NC_000017.11:c41530308-41528309	1980	1992	-	CCTCACCTGGCGC
MA0830.2	MA0830.2.TCF4	9.73098	0.8685529996825981	NC_000017.11:c41530308-41528309	755	767	-	TAGCATCTGCCAC
MA0830.2	MA0830.2.TCF4	9.644816	0.8668070693038972	NC_000017.11:c41530308-41528309	1824	1836	-	AGGCCCCTGCCCT

Supplementary Figure S5 Prediction of potential targets of TCF4 using the JASPAR database

In the JASPAR database, the potential regulatory effect of TCF4 on the transcription of *Sox9*, *Alb*, *Hnf4a*, and *Ck19* was predicted.

Supplementary tables

Supplementary Table S1. Clinical characteristics of patients with liver cirrhosis and normal controls

Group	Age	Gender	Diagnosis	HBV	Sample
Normal 1	57	F	Normal	-	Liver tissue
Normal 2	26	F	Normal	-	Liver tissue
Normal 3	42	M	Normal	-	Liver tissue
Normal 4	69	F	Normal	-	Liver tissue
Normal 5	51	F	Normal	-	Liver tissue
Normal 6	49	F	Normal	-	Liver tissue
Normal 7	23	M	Normal	-	Liver tissue
Cirrhosis 1	79	M	Cirrhosis	-	Liver tissue
Cirrhosis 2	54	M	Cirrhosis	+	Liver tissue
Cirrhosis 3	70	M	Cirrhosis	+	Liver tissue
Cirrhosis 4	57	M	Cirrhosis	+	Liver tissue
Cirrhosis 5	39	M	Cirrhosis	-	Liver tissue
Cirrhosis 6	56	M	Cirrhosis	-	Liver tissue
Cirrhosis 7	50	M	Cirrhosis	+	Liver tissue
Cirrhosis 8	58	M	Cirrhosis	+	Liver tissue
Cirrhosis 9	53	M	Cirrhosis	-	Liver tissue
Cirrhosis 10	60	M	Cirrhosis	-	Liver tissue
Cirrhosis 11	69	M	Cirrhosis	-	Liver tissue

Supplementary Table S2. Antibodies used in this study

Antibody	Application	Dilution	Source	Company and cat no
**Non-phospho (Active) β-Catenin**	IF<br>WB	1:1000	Rabbit	Cell Signaling Technology #8814
**Total β-catenin**	WB	1:1000	Rabbit	Cell Signaling Technology #8480
**Cyclin D1**	IF<br>WB	1:200<br>1:2000	Rabbit	Huabio #ET1601-31
**ALB**	IF<br>WB	1:200<br>1:5000	Mouse<br>Rabbit	Proteintec #66051-1-Ig<br>Proteintec #16475-1-AB
**HNF4α**	IF<br>WB	1:200<br>1:5000	Mouse	Invitrogen #MAI-199
**CK19**	IF<br>IHC<br>WB	1:500<br>1:200<br>1:2000	Rabbit	Huabio #ER1803-79
**SOX9**	IF<br>WB	1:200<br>1:1000	Rabbit	Huabio #ET1611-56
**COX-2**	WB	1:2000	Rabbit	Abcam #ab15191
**TGFBR1**	WB	1:1000	Rabbit	Abcam #ab235578
**BSEP**	IF	1:100	Mouse	Santa Cruz Biotechnology #Sc-74500
**ZO-1**	IF	1:500	Rabbit	Proteintech #21773-1-AP
**GAPDH**	WB	1:10000	Mouse	Abclonal #AC033

IF: Immunofluorescence; IHC: Immunohistochemistry; WB: Western blot; ALB: albumin; HNF4α: hepatocyte nuclear factor 4 alpha; CK19: cytokeratin 19; SOX9: SRY-box transcription factor 9; COX-2: cyclooxygenase-2; TGFBR1: transforming growth factor beta receptor 1; BSEP: bile salt export pump; GAPDH: glyceraldehyde-3-phosphate dehydrogenase.

Supplementary Table S3. qPCR primers

Gene	Sequence-forward	Sequence-reverse
**Primers for mouse qRT-PCR**		
Abcb11	TCTGACTCAGTGATTCTT CGCA	CCCATAAACATCAGCCAGTTG T
Slc10a1	CAAACCTCAGAAGGACC AAACA	GTAGGAGGATTATTCCCGTTG TG
Abcc3	CTGGGTCCCCTGCATCTA C	GCCGTCTTGAGCCTGGATAAC
Slc51b	AGATGCGGCTCCTTGGAA TTA	TGGCTGCTTCTTTCGATTTCTG
Nr1h4	TGGGCTCCGAATCCTCTT AGA	TGGTCCTCAAATAAGATCCTT GG
Nr0b2	CAGGTCGTCCGACTATTC TGT	AGGCTACTGTCTTGGCTAGGA
Gpbar1	GTCAGCTCCCTGTTCTTT GC	CAGGAGGCCATAAACTTCCA
Fgf15	ATGGCGAGAAAGTGGAA CGG	GGACCAGCGGAGTACAGGT
Cyp7a1	AAACTCCCTGTCATACCA CAAAG	TTTCCATCACTTGGGTCTATG C
Cyp7b1	GGAGCCACGACCCTAGAT G	GCCATGCCAAGATAAGGAAG C
Cyp8b1	AGTACACATGGACCCCGA CATC	GGGTGCCATCCGGGTTGAG
Cyp27a1	GCCTCACCTATGGGATCT TCA	TCAAAGCCTGACGCAGATG
Tgfbr1	TCTGCATTGCACTTATGCT GA	AAAGGGCGATCTAGTGATGG A
β-actin	TGACGTTGACATCCGTAA AG	GAGGAGCAATGATCTTGATCT
**Primers for mouse genotyping-PCR**		
Alb-Cre	CATATTGGCAGAACGAAA ACGC	CCTGTTTCACTATCCAGGTTA CGG
Ella-Cre	CATATTGGCAGAACGAAA	CCTGTTTCACTATCCAGGTTA

ACGC	CGG

Abcb11: ATP binding cassette subfamily B member 11; *Slc10a1*: solute carrier family 10 member 1; *Abcc3*: ATP binding cassette subfamily C member 3; *Slc51b*: solute carrier family 51 subunit beta; *Nr1h4*: nuclear receptor subfamily 1 group H member 4; *Nr0b2*: nuclear receptor subfamily 0 group B member 2; *Gpbar1*: G protein-coupled bile acid receptor 1; *Fgf15*: fibroblast growth factor 15; *Cyp7a1*: cytochrome P450 family 7 subfamily A member 1; *Cyp7b1*: cytochrome P450 family 7 subfamily B member 1; *Cyp8b1*: cytochrome P450 family 8 subfamily B member 1; *Cyp27a1*: cytochrome P450 family 27 subfamily A member 1; *Tgfbr1*: transforming growth factor beta receptor 1.
