# Supplementary material for: Atypical cholangiocytes derived from hepatocyte-cholangiocyte transdifferentiation mediated by COX-2: a kind of misguided liver regeneration
Source: Inflamm Regen. 2023 Jul 14;43:37. doi: 10.1186/s41232-023-00284-4 (PMC10347763; doi:10.1186/s41232-023-00284-4)

Figure 1e

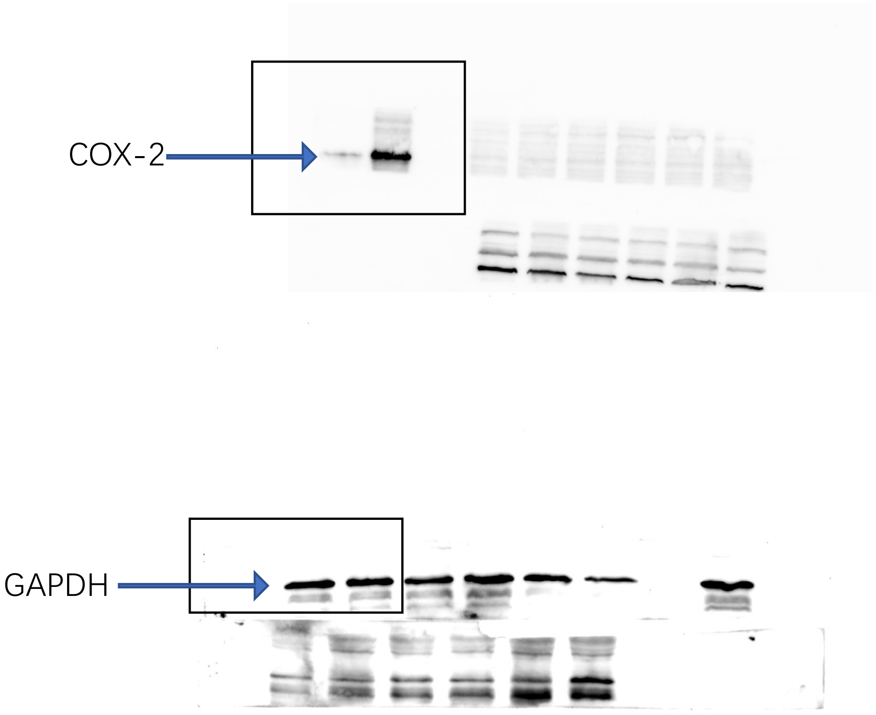

Figure 4d

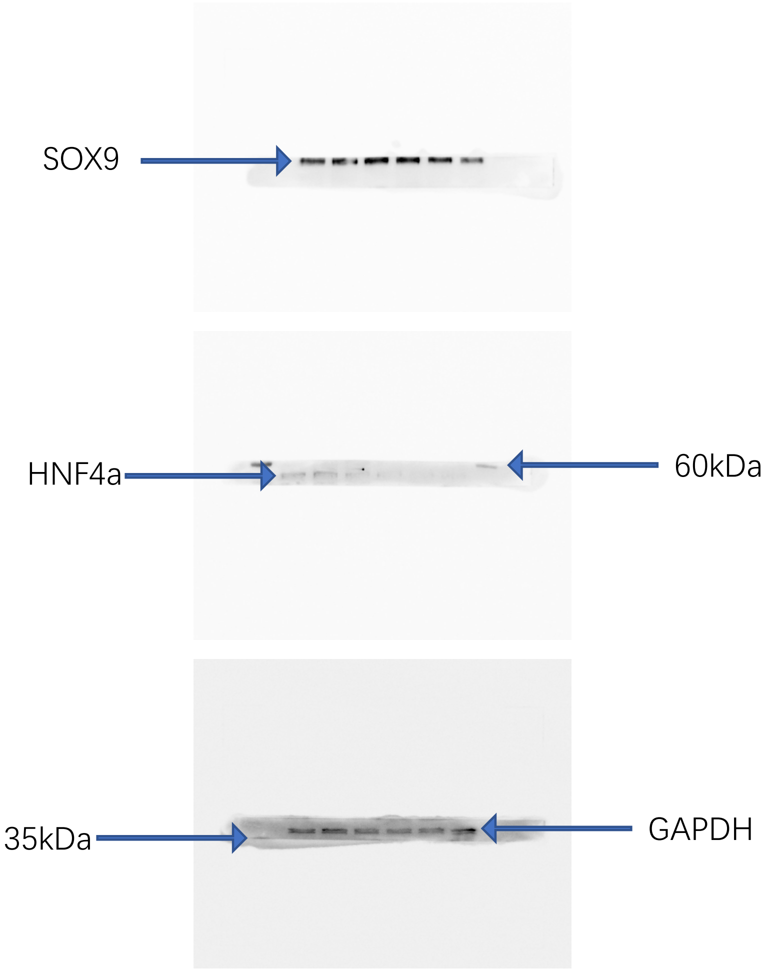

Figure 5b

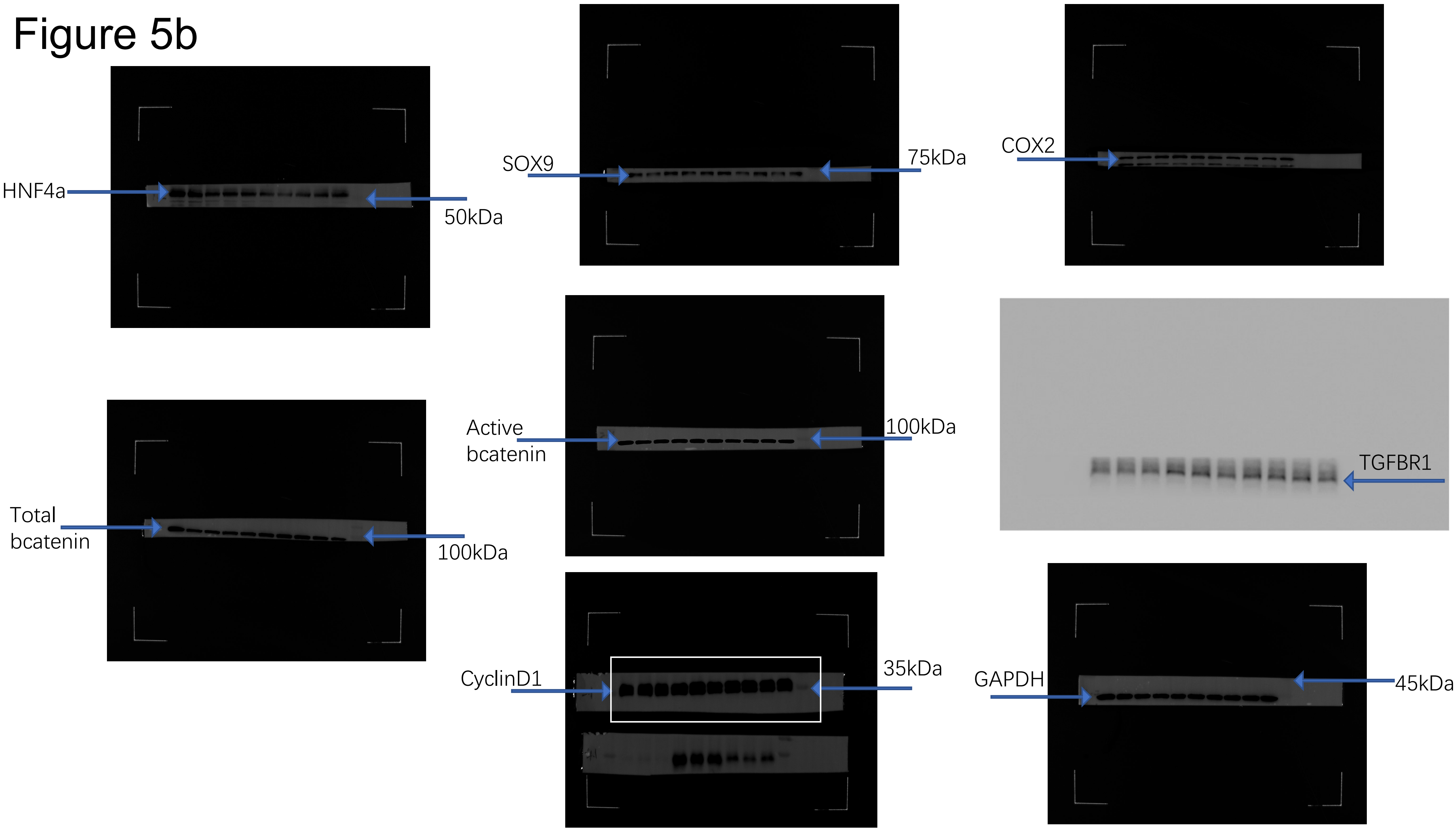
HNF4a
50kDa
SOX9
75kDa
COX2
Total
bcatenin
100kDa
Active
bcatenin
100kDa
TGFBR1
CyclinD1
35kDa
GAPDH
45kDa

Figure 6a

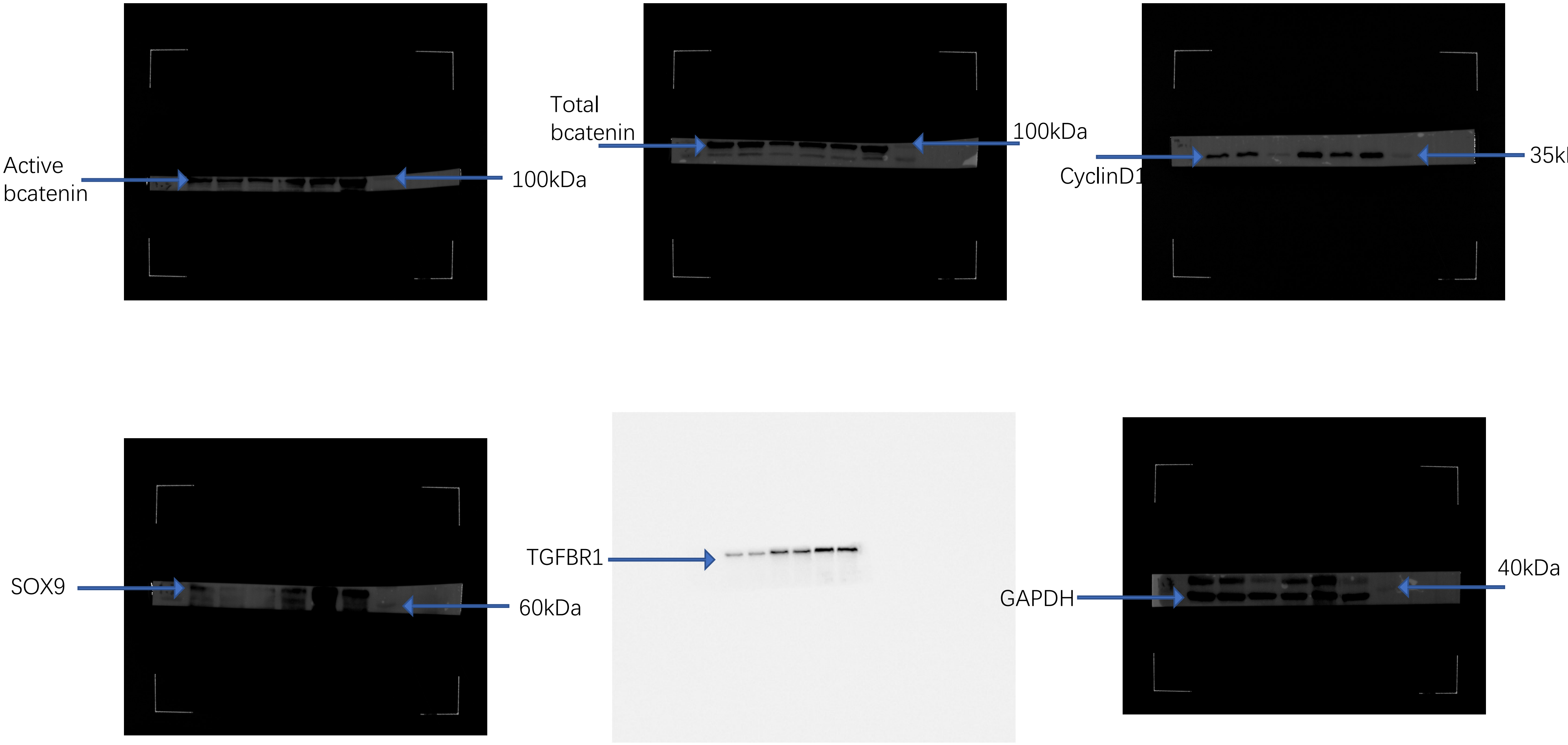

Active
bcatenin
100kDa
Total
bcatenin
100kDa
CyclinD1
35k
SOX9
60kDa
TGFBR1
GAPDH
40kDa

Figure 6b

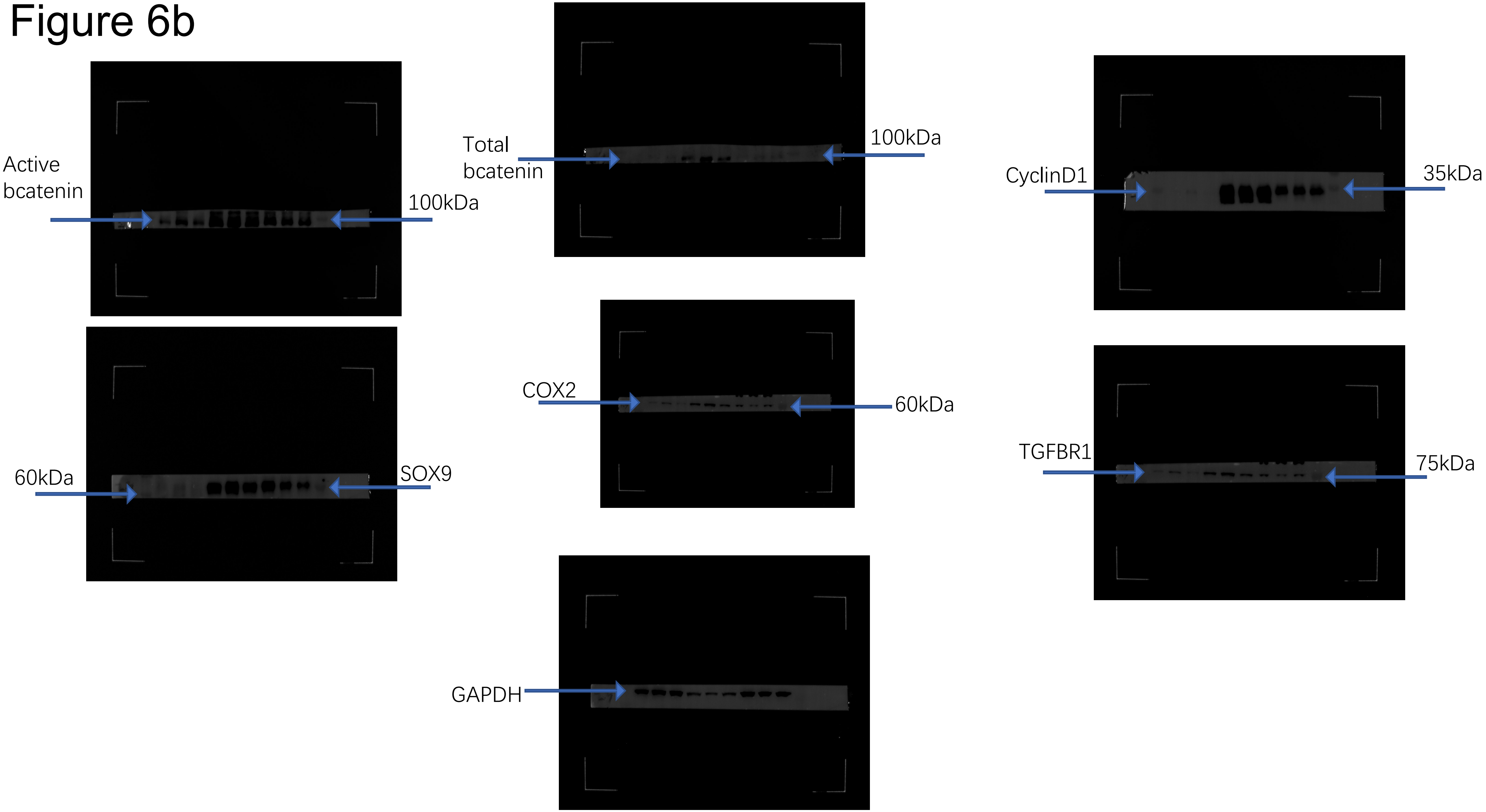

Active bcatenin
100kDa
Total bcatenin
100kDa
CyclinD1
35kDa
60kDa
SOX9
COX2
60kDa
TGFBR1
75kDa
GAPDH

Figure 7a

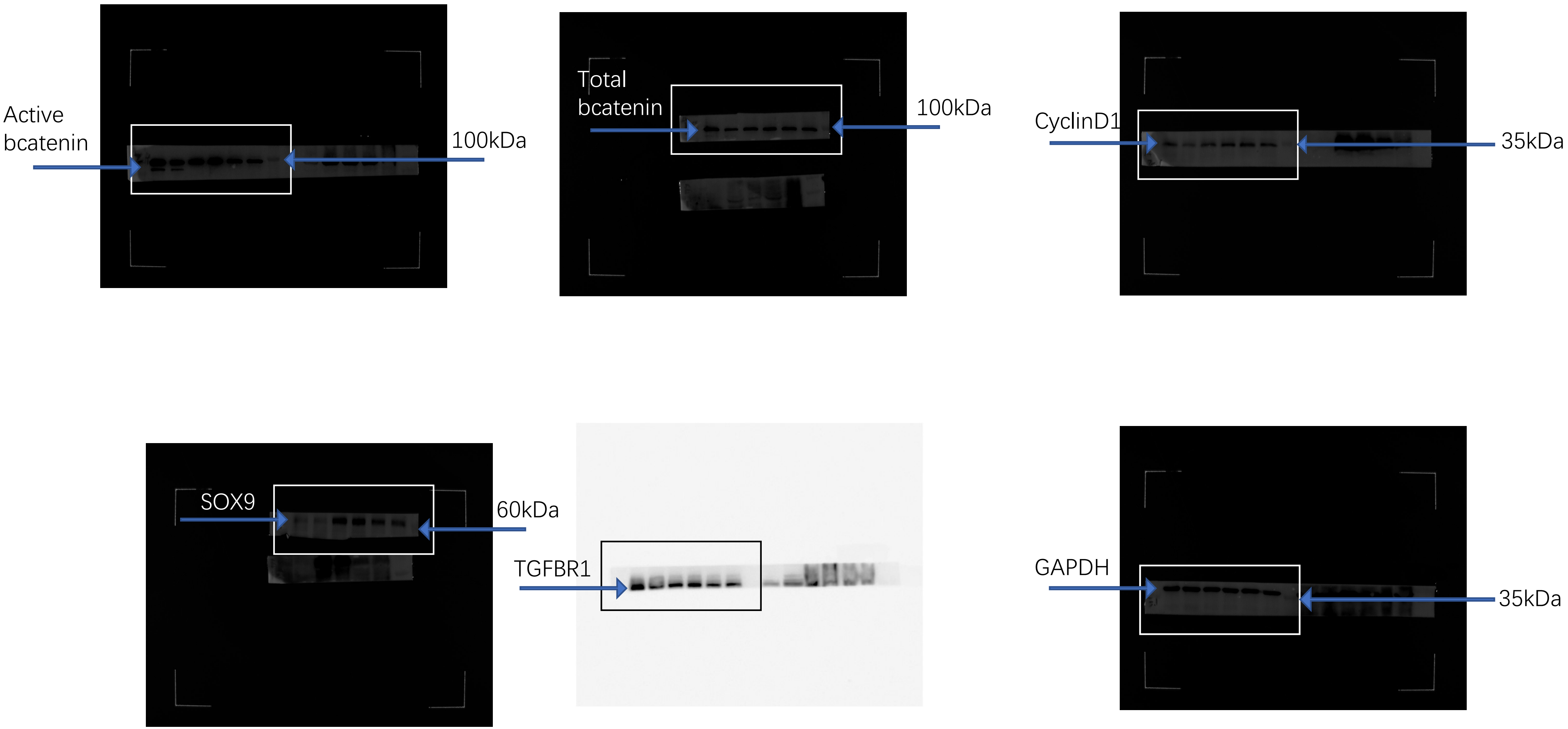

Active
bcatenin
100kDa
Total
bcatenin
100kDa
CyclinD1
35kDa
SOX9
60kDa
TGFBR1
GAPDH
35kDa

Figure 7d

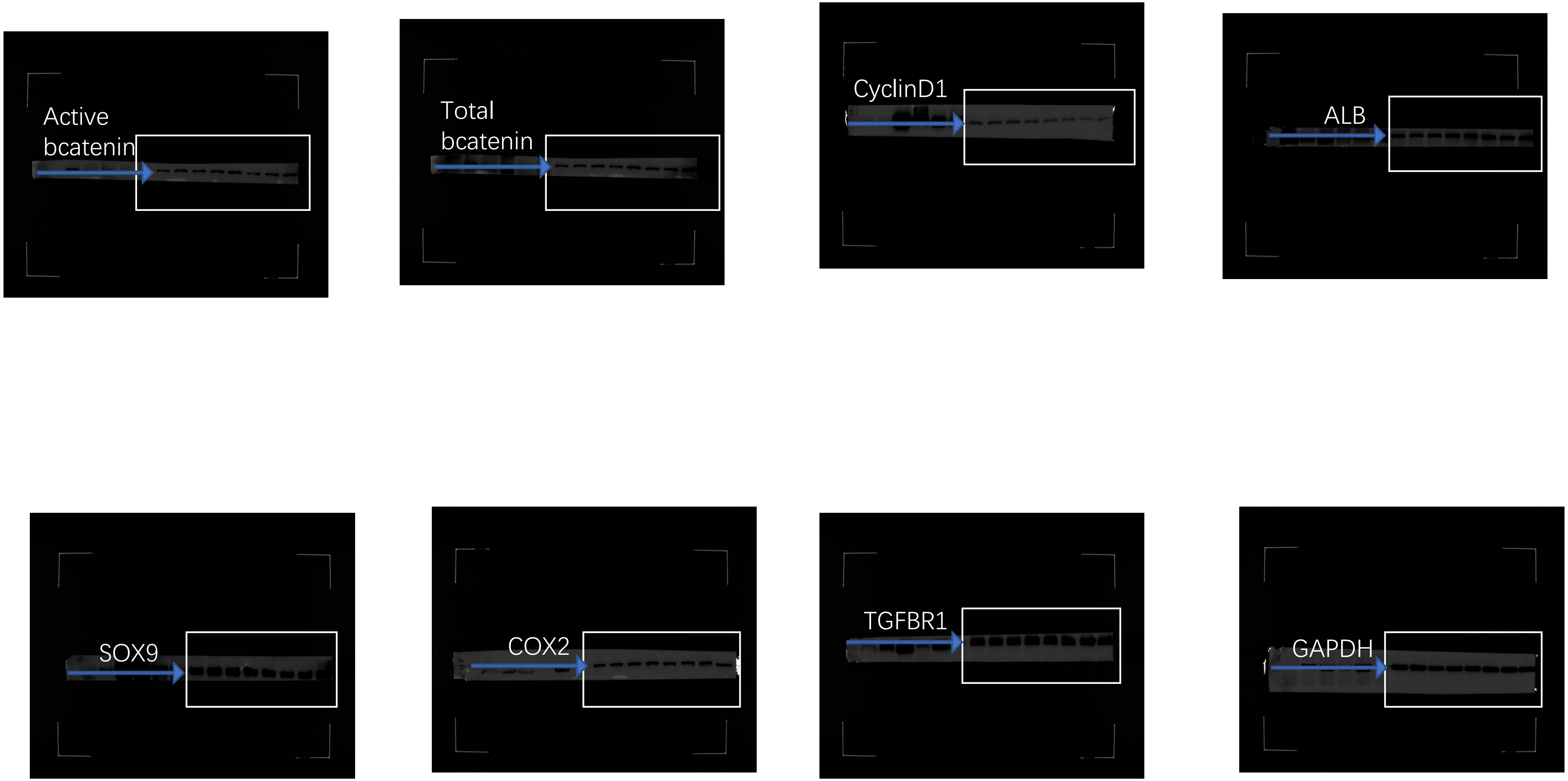
Active bcatenin
Total bcatenin
CyclinD1
ALB
SOX9
COX2
TGFBR1
GAPDH

Supplementary Figure S3a

Active bcatenin

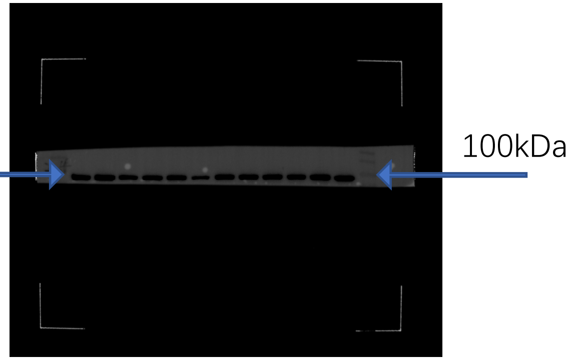

100kDa

SOX9

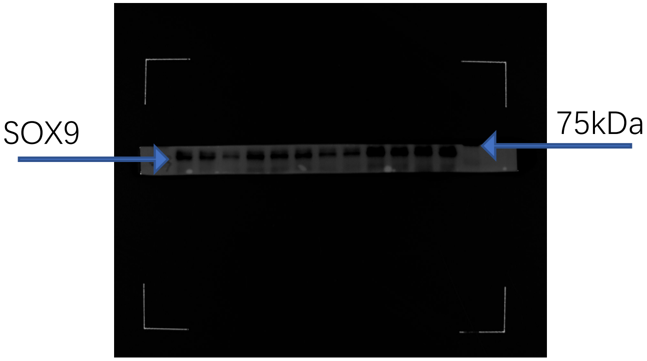

75kDa

Total bcatenin

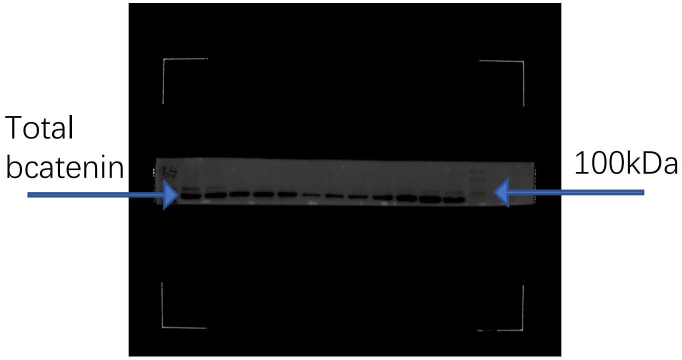

100kDa

CK19

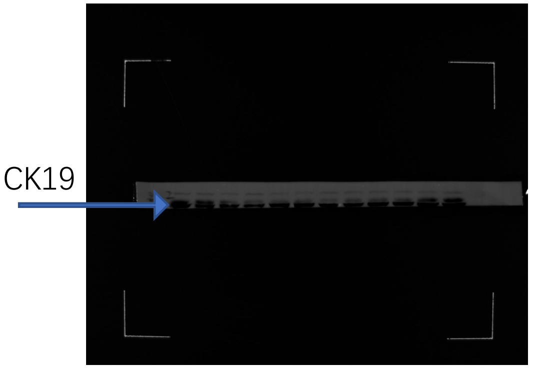

GAPDH

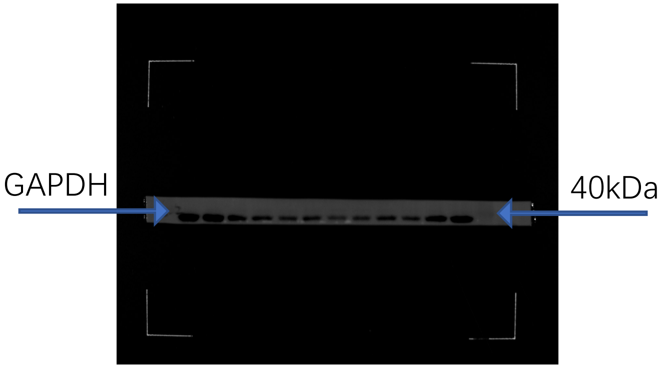

40kDa

CyclinD1

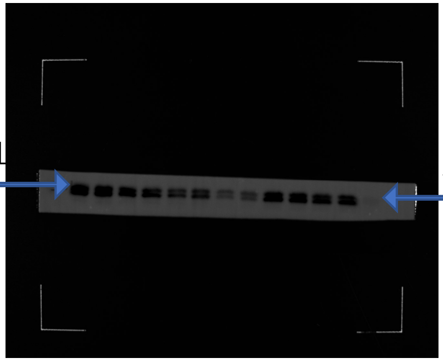

35kDa

COX2

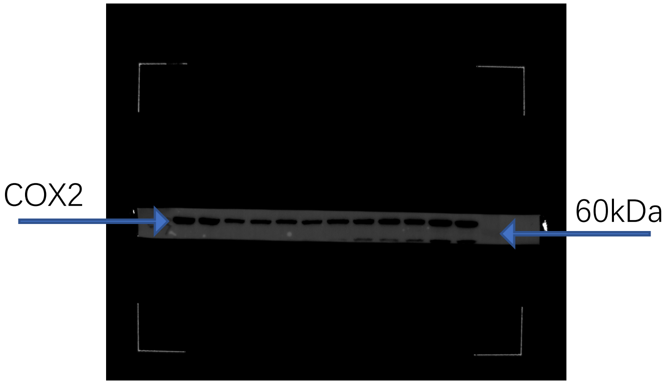

60kDa

Supplementary Figure S3c

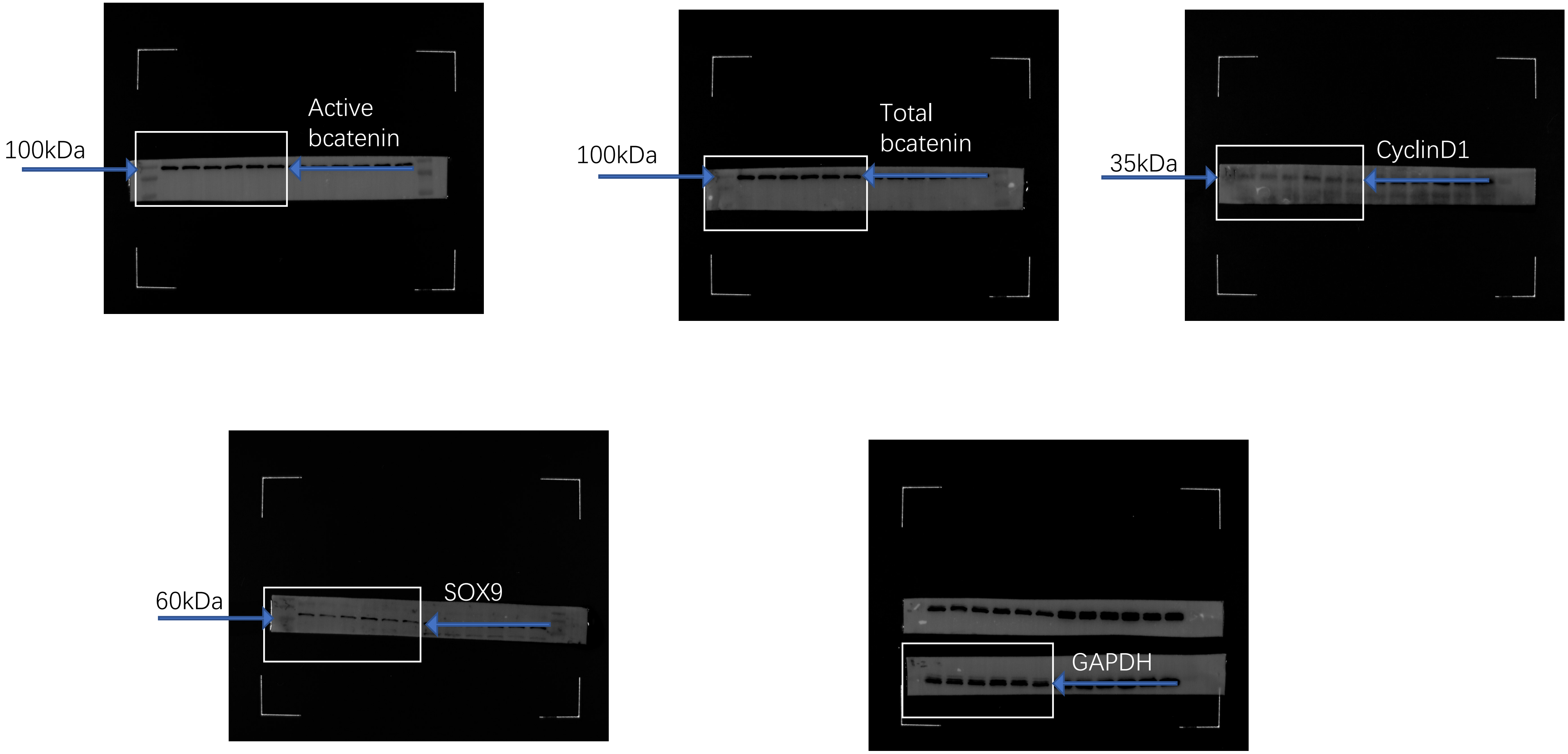

Supplement: Supplementary file 2 — Additional file 2. Original WB data. [file 41232_2023_284_MOESM2_ESM.pdf]
